# Vitrification and Rewarming of Magnetic Nanoparticle-Loaded Rat Hearts

**DOI:** 10.1002/admt.202100873

**Published:** 2021-10-01

**Authors:** Zhe Gao, Baterdene Namsrai, Zonghu Han, Purva Joshi, Joseph Sushil Rao, Vasanth Ravikumar, Anirudh Sharma, Hattie L. Ring, Djaudat Idiyatullin, Elliott C. Magnuson, Paul A. Iaizzo, Elena G. Tolkacheva, Michael Garwood, Yoed Rabin, Michael Etheridge, Erik B. Finger, John C. Bischof

**Affiliations:** Department of Mechanical Engineering, University of Minnesota, 111 Church St. SE., Minneapolis, MN 55455, USA; Department of Surgery, University of Minnesota, 420 Delaware Street SE, Minneapolis, MN 55455, USA; Department of Mechanical Engineering, University of Minnesota, 111 Church St. SE., Minneapolis, MN 55455, USA; Department of Mechanical Engineering, Carnegie Mellon University, 5000 Forbes Avenue, Pittsburgh, PA 15213, USA; Department of Surgery, University of Minnesota, 420 Delaware Street SE, Minneapolis, MN 55455, USA; Department of Biomedical Engineering, University of Minnesota, 312 Church St. SE, Minneapolis, MN 55455, USA; Department of Mechanical Engineering, University of Minnesota, 111 Church St. SE., Minneapolis, MN 55455, USA; Center for Magnetic Resonance Research, Department of Radiology, University of Minnesota, 2021 6th Street S.E. Minneapolis, Minneapolis, MN 55455, USA; Center for Magnetic Resonance Research, Department of Radiology, University of Minnesota, 2021 6th Street S.E. Minneapolis, Minneapolis, MN 55455, USA; Department of Mechanical Engineering, University of Minnesota, 111 Church St. SE., Minneapolis, MN 55455, USA; Department of Surgery, University of Minnesota, 420 Delaware Street SE, Minneapolis, MN 55455, USA; Department of Biomedical Engineering, University of Minnesota, 312 Church St. SE, Minneapolis, MN 55455, USA; Center for Magnetic Resonance Research, Department of Radiology, University of Minnesota, 2021 6th Street S.E. Minneapolis, Minneapolis, MN 55455, USA; Department of Mechanical Engineering, Carnegie Mellon University, 5000 Forbes Avenue, Pittsburgh, PA 15213, USA; Department of Mechanical Engineering, University of Minnesota, 111 Church St. SE., Minneapolis, MN 55455, USA; Department of Surgery, University of Minnesota, 420 Delaware Street SE, Minneapolis, MN 55455, USA; Department of Mechanical Engineering, University of Minnesota, 111 Church St. SE., Minneapolis, MN 55455, USA; Department of Surgery, University of Minnesota, 420 Delaware Street SE, Minneapolis, MN 55455, USA; Department of Biomedical Engineering, University of Minnesota, 312 Church St. SE, Minneapolis, MN 55455, USA

**Keywords:** cryopreservation, heart, iron oxide nanoparticle, radio frequency warming, vitrification

## Abstract

To extend the preservation of donor hearts beyond the current 4–6 h, this paper explores heart cryopreservation by vitrification—cryogenic storage in a glass-like state. While organ vitrification is made possible by using cryoprotective agents (CPA) that inhibit ice during cooling, failure occurs during convective rewarming due to slow and non-uniform rewarming which causes ice crystallization and/or cracking. Here an alternative, “nanowarming”, which uses silica-coated iron oxide nanoparticles (sIONPs) perfusion loaded through the vasculature is explored, that allows a radiofrequency coil to rewarm the organ quickly and uniformly to avoid convective failures. Nanowarming has been applied to cells and tissues, and a proof of principle study suggests it is possible in the heart, but proper physical and biological characterization especially in organs is still lacking. Here, using a rat heart model, controlled machine perfusion loading and unloading of CPA and sIONPs, cooling to a vitrified state, and fast and uniform nanowarming without crystallization or cracking is demonstrated. Further, nanowarmed hearts maintain histologic appearance and endothelial integrity superior to convective rewarming and indistinguishable from CPA load/unload control hearts while showing some promising organ-level (electrical) functional activity. This work demonstrates physically successful heart vitrification and nanowarming and that biological outcomes can be expected to improve by reducing or eliminating CPA toxicity during loading and unloading.

## Graphical Abstract

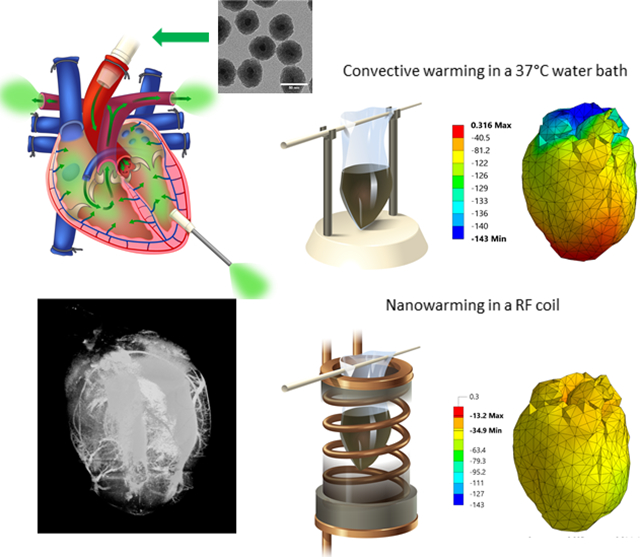

Heart cryopreservation by vitrification and nanowarming has immense promise for transforming transplant medicine. Here we show comprehensive validation of the approach, including machine perfusion loading/unloading of cryoprotective agents (CPA) and magnetic nanoparticles, cooling to a vitrified state, and fast and uniform radiofrequency rewarming without crystallization or cracking. Nanowarmed hearts maintained histologic appearance and endothelial integrity similar to CPA load/unload controls with some organ-level (electrical) functional activity.

## 1. Introduction

Today, heart transplantation remains the treatment of choice for patients with end-stage heart failure. However, the feasibility of transplantation is limited in part, by the availability of suitable donor organs. In the United States, 17% of heart transplant candidates die before a suitable donor heart becomes available,^[1]^ and the shortage of donor hearts is even more pronounced throughout the rest of the world.^[2]^ The current gold standard method for preserving hearts is static cold storage, where the donor heart is preserved in an organ preservation solution on ice for 4–6 h.^[3]^ To extend the duration of preservation, many approaches have been attempted, such as continuous cold perfusion at 4–8 °C, which achieved functional recovery in rabbit hearts after 24 h of perfusion with modified University of Wisconsin (UW) solution as perfusate^[4]^ and the maintenance of left ventricle (LV) function after 24 h of perfusion with other additives.^[5]^ Indeed, Rosenfeldt et al. reported good functional and metabolic recovery after 12 h of cold perfusion in a deceased human heart.^[6]^ Another promising technology is normothermic extracorporeal machine perfusion, in which oxygenated blood or blood substitute is perfused through the heart at normothermic temperature.^[1b,7]^ The TransMedics Organ Care System, OCS Heart, is recently approved by FDA for use in the normothermic perfusion of donor hearts, which may extend the heart storage time to 10 h^[8]^ and also re-condition donor hearts that arrive in marginal condition.^[9]^ However, no heart storage method is currently able to extend the duration of preservation beyond 1–2 d, let alone weeks, months, or years.^[10]^

Fortunately, the longer-term storage of cells and tissues can be achieved through cryopreservation, albeit not yet for organs. In this approach, the biological systems are first loaded with a cryoprotective agent (CPA, usually alcohols, sugars, polymers, and/or ice blockers) and then cooled in a way that either controls ice formation or avoids ice crystallization altogether. Vitrification (cooling to a glassy phase without ice crystal formation) has been championed as one, if not the only, long-term solution for organ cryopreservation.^[11]^ Although vitrification after cooling has been achieved in cell, tissue, and whole organ systems since the 1980s,^[11,12]^ rewarming these materials from the cryopreserved state remains a challenge for larger systems such as organs (>3 mL).^[13]^ The source of this failure is related to the limitations of conventional convective rewarming (e.g., warming in a controlled rate freezer or immersion in a water bath) in which heat can be applied on only the boundary of the system, thereby leading either to excessively low rates which favor crystallization or fast rates with large temperature gradients between surface and center which induces thermal-mechanical stress that can drive catastrophic cracking (i.e., when an ice cube cracks when added to water).^[14]^ It is therefore not surprising that multiple studies on frozen state cryopreservation of rat hearts show that ice formation causes irreparable structural damage to the hearts and fails to restore function.^[10a,15]^

Recently, our group and others have explored the use of magnetic nanoparticle enabled volumetric heating entitled “nanowarming” for use in bulk rewarming of cryopreserved biomaterials including cells, tissues, and organs.^[13,16]^ In nanowarming, magnetic nanomaterials such as iron oxide nanoparticles (IONPs) can be preloaded into the sample along with CPA before cooling to a vitrified state. The vitrified specimen can then be rewarmed in a radio frequency (RF) coil (i.e., alternating magnetic field), where the IONPs are excited by magnetic hysteresis thereby generating rapid and uniform heating within the sample that can overcome convective (boundary) warming, which fails by crystallization and/or cracking of the sample.^[17]^

Nanowarming was originally achieved using IONPs, but other nanomaterials such as nanowires,^[16a]^ larger core,^[18]^ or cubic IONPs^[19]^ can in theory also be used for rewarming hearts or other tissues and organs as they are excellent heaters (1000s W g^−1^ Fe). Nevertheless, the size and aspect ratio of some of these nanomaterials make them less colloidally stable (i.e., they crash out of solution). Colloidal stability in particular is a key property as these nanomaterials must perfuse into and out of organs and remain stable in CPA suspensions.^[13,16b]^ One key to colloidal stability is the particle coating which may consist of PEG coated silica (msIONP,^[13]^ silica-coated iron oxide nanoparticle [sIONP]^[16b]^) or PEG,^[16c]^ both of which also confer biocompatibility (i.e., reduces cellular uptake). IONPs are generally considered biocompatible and have been widely used in imaging, drug delivery, and treatment of various disorders.^[20]^ IONPs have also been used in cardiac systems and shown low cardiomyocyte toxicity,^[21]^ some protection from ischemic damage,^[22]^ and ability to treat heart infarcts.^[21a,23]^ Finally, a proof-of-principle study in heart was recently published suggesting the potential feasibility of heart nanowarming.^[16c]^ These rodent organs will require 100s of mg of magnetic materials, while nanowarming of larger animal and human organs will require grams of Fe/organ,^[16b]^ so the synthesis of these materials also needs to be scalable.

For these reasons, smaller iron oxide nanoparticles have been the magnetic material of choice for most nanowarming applications.^[13,16a–c]^ Foremost among these, sIONPs showed high heating, high colloidal stability in CPAs, and scalable production.^[16b]^ These sIONPs have been thoroughly characterized by transmission electronic microscopy (TEM), dynamic light scattering (DLS), zeta potential, inductively coupled plasmaoptical emission spectroscopy (ICP-OES), infrared spectroscopy (IR), X-ray photoelectron spectroscopy (XPS), nitrogen adsorption analysis, and thermal gravity analysis (TGA) (Figure S1, Supporting Information, and as previously reported^[16b]^). Finally, these sIONPs showed minimal cellular interaction with human dermal fibroblast and show good washout from rat kidneys after loading^[16b]^ and hence were selected as the IONP of choice for this study on heart nanowarming.

Based on our preliminary findings using nanowarming for the successful cryopreservation of vascular grafts, and supported by the proof-of-principle heart nanowarming work of another group,^[16c]^ we sought to determine if whole hearts could be cryopreserved for potential future regenerative medicine or transplantation applications. Notable new physical and biological data in the current work versus the previous work^[16c]^ include: 1) quantification of CPA and sIONP loading and washout after machine perfusion (vs syringe injection), 2) achievement of successful cooling and vitrification by advanced imaging (microcomputed tomography [μCT]), 3) achievement of sufficiently fast and uniform rewarming to avoid crystallization and cracking by advanced thermometry and modeling, and 4) biological (metabolic, histological, and electrophysiological) evidence of viability and function post re-rewarming. Further, based on our new data showing the equivalency of CPA loaded and unloaded groups to nanowarmed groups, and the first demonstration of electrical activity in a nanowarmed heart, further improvements in CPA and loading/ unloading procedures suggest nanowarming and functional recovery of whole mammalian hearts is an achievable goal.

## 2. Results and Discussion

**Figure 1.**
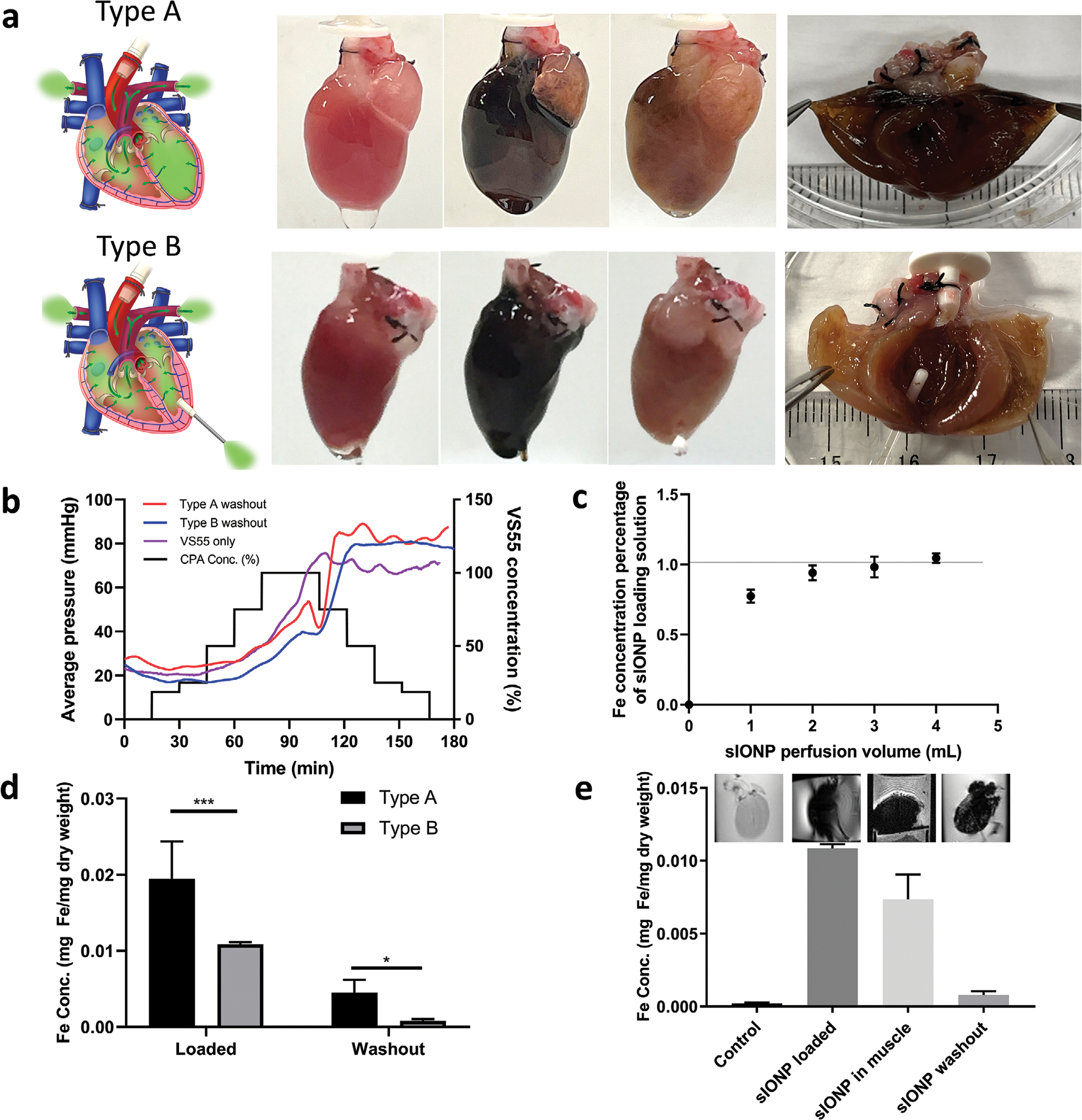
Impact of heart cannulation method on VS55 and sIONP loading and unloading in hearts. a) Schemes of type A and type B cannulation methods. Photos of hearts from left to right: pre-sIONP loading, post-sIONP loading, and post-sIONP washout and a cut open heart post-sIONP washout. b) Average perfusion resistance pressure of type A and B hearts during sIONP loading and removal and of a type B heart during VS55 loading and removal, and the VS55 concentration of the VS55 loading steps. *n* = 3. c) Fe concentration in the effluent from the heart compared to the loaded sIONP Fe concentration. *n* = 3. d) Fe concentration of loaded (*p* < 0.001) and washout (*p* = 0.0181) type A and type B hearts. Type A loaded *n* = 3, Type A washout *n* = 6, Type B loaded *n* = 5, Type B washout *n* = 5. e) Fe concentration in control heart, sIONP-loaded heart, sIONP in muscle, and sIONP washed-out type B heart. Control *n* = 7, sIONP loaded *n* = 5, sIONP in muscle *n* = 3, sIONP washout *n* = 5. The upper images are the corresponding T2*-weighted GRE MRI images. The dark lines in the image are the Teflon scaffold used to secure the heart. *p* < 0.0001 for all group comparison except control versus washout.

The perfusion of VS55 in a kidney has been previously reported by Fahy et al.,^[24]^ and most recently syringe injection of VS55 in a heart was reported by Chiu-Lam et al.^[16c]^ However, no group has shown controlled VS55 machine perfusion, including resistance and distribution measurements, in intact hearts followed by cryogenic storage and rewarming. In the current study, VS55 was perfused into a rat heart in a step-wise fashion to reduce osmotic shock (Figure 1). When the perfusate reached 100% VS55, a new stream of 100% VS55 loaded with 10 mg Fe mL^−1^ sIONPs^[16b]^ (Figure S1, Supporting Information) was perfused into the heart until nanoparticle saturation was achieved in the effluent. The heart was then placed into a polyethylene cryobag containing 10 mL of sIONP/VS55 solution, vitrified in a controlled rate freezer by cooling at −40 °C min^−1^ to −150 °C with an annealing step at −122 °C, and transferred to long-term storage (at −150 °C). Nanowarming was performed in a 15 kW 80 mL RF coil.^[13]^ The rewarmed heart was then re-perfused with sequentially lower concentrations of VS55 in a step-wise fashion to remove VS55 and sIONPs (Figure 1).

The sIONPs were synthesized as we have previously reported.^[16b]^ Briefly, each batch of sIONPs was characterized by TEM, DLS, ICP-OES, and a heating generation (specific absorption rate [SAR]). Representative TEM images and hydrodynamic diameter of sIONPs are shown in Figure S1 (Supporting Information). A uniform 16 nm-thick silica shell was coated on the EMG308 core. The silica shell as well as the surface modification provide colloidal stability in highly viscous and salt laden CPAs such as VS55. Moreover, sIONP were colloidally stable in both VS55 and M22 at 10 mg Fe mL^−1^ for over four months (Figure S1c, Supporting Information). An added benefit of these particles is that the silica coating acts as a steric spacer between the IONP cores, making sIONPs less affected by inter-particle interactions at high concentration and allowing relatively high heating in complex solutions such as CPAs versus water (360 vs 400 W g^−1^ Fe).^[16b]^ Finally, we have found that sIONPs demonstrate little, if any, cellular interaction/uptake and most of the sIONPs loaded in rat kidneys are cleared during unloading. With this background we used sIONP for loading, distribution, heat generation, and washout in rat hearts as described below.

### 2.1. Influence of Cannulation Method on sIONP Distribution and Washout

The Langendorff method is a standard approach for cardiac perfusion that enables the delivery of oxygen and nutrients to the myocardium, thereby facilitating the study of cardiac physiology, such as contractile function, and coronary blood flow.^[25]^ In brief, perfusate was delivered to the heart through a cannula inserted in the ascending aorta and thus into the coronary arteries. After passing through the coronary system, the perfusate drained into the right atrium (RA) via the coronary sinus (Figure S2, Supporting Information).

However, this method has limitations. For instance, as pressure rises in the cannulated aorta the aortic valve can close, thereby allowing perfusate to enter the coronary arteries via the ostia at the aortic root. While the bulk of flow drains into the right heart via the coronary sinus and eventually leaves via the pulmonary artery, some directly enters the atria and ventricles via Thebesian vessels. Note, that since the aortic valve remains closed, there is no outflow from the left side of the heart and as pressure begins to rise the LV can become distended (Figure 1a).^[26]^

To reduce the pressure buildup in the LV and support sIONP drainage, a catheter was inserted into the LV to act as a vent (Figure 1a). To prepare for future heterotopic intra-abdominal heart transplantation, pulmonary veins (PV), inferior vena cava (IVC), and superior vena cava (SVC) were ligated.^[27]^ Herein, type A refers to a heart cannulated by the Langendorff method, and type B refers to a heart cannulated by the modified Langendorff method with an added LV vent. In addition to the type A and type B approaches, we also tested placing a catheter in the left heart via the left atrial appendage and tested disruption of the mitral valve and open drainage of the LA with gravity drainage. Both of these latter approaches were unsuccessful in washing out the sIONPs from the LV and were abandoned (Figure S3, Supporting Information).

#### 2.1.1. Perfusion Resistance

A constant flow rate with continuous pressure monitoring was used to deliver perfusate and assess the perfusion resistance in our study.^[28]^ The VS55 solution was introduced into the hearts in a step-wise manner to reduce osmotic shock, as previously described.^[12]^ After sIONP loading, the hearts were unloaded in a step-wise fashion with dilutions of VS55. Both loading and removal steps were kept the same for both cannulation types, as shown in Figure 1b. Of note, the perfusion pressures during VS55 removal were consistently higher than seen during loading regardless of whether sIONPs had been added. This was likely due to cell swelling and reduced vascular diameter during washout with relatively hypotonic perfusate as concentration returns from high concentration to normotonic media. The relative changes in observed resistance were consistent with prior Krogh cylinder modeling of perfusion with increasing and decreasing relative tonicity solutions.^[29]^

#### 2.1.2. sIONP Distribution in sIONP-Loaded Heart

**Figure 2.**
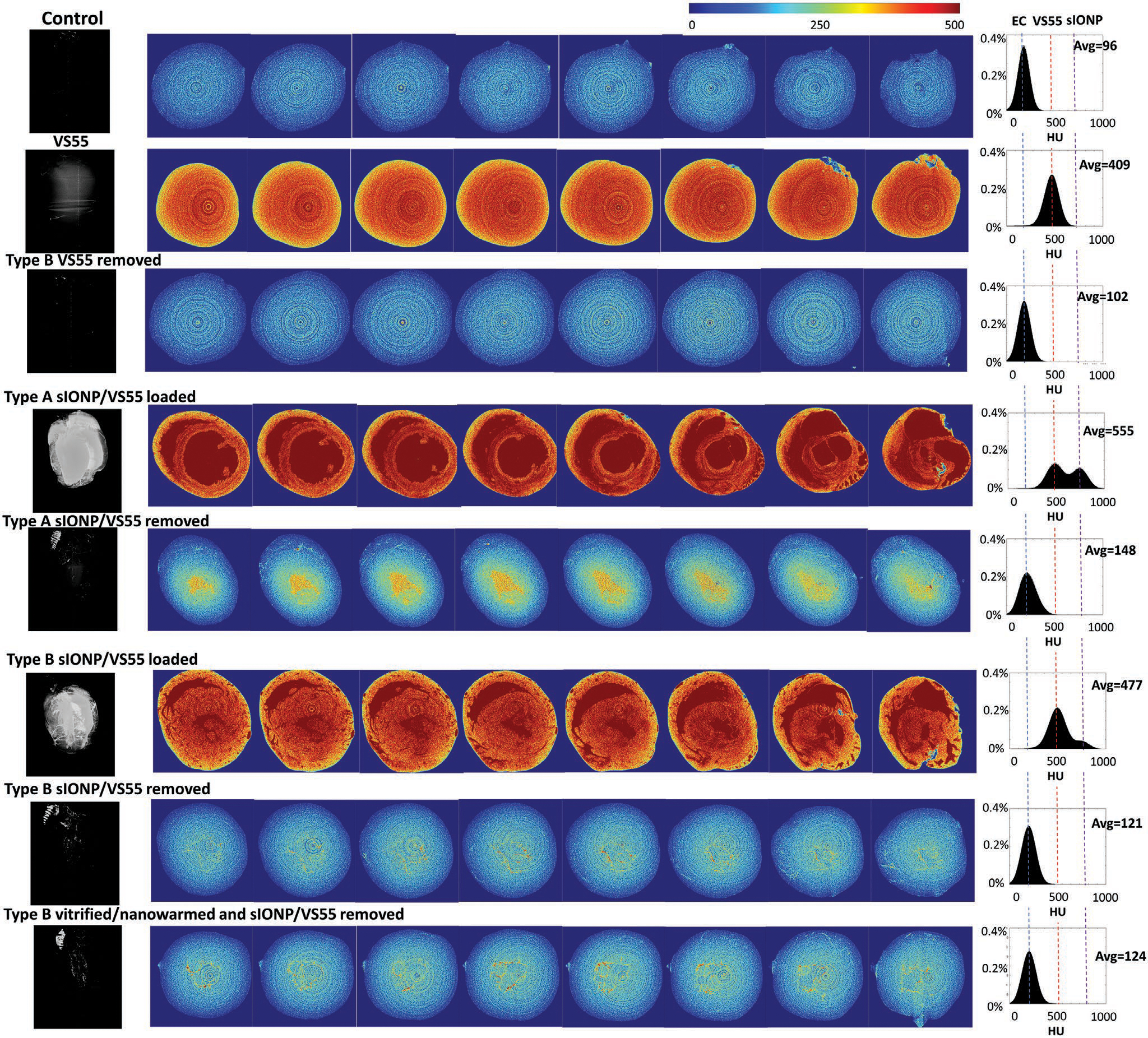
Microcomputed tomography (μCT) images of experimental and control heart groups. From top to bottom: a control heart perfused with EC, a heart perfused with VS55, A type B heart after VS55 removal, a type A heart perfused with VS55 and sIONP, a type A heart after removal steps, a type B heart perfused with VS55 and sIONP, a type B heart after removal steps, and a type B heart after vitrification/nanowarming and removal steps. The left column is μCT images of hearts in HU 500–800 indicating the sIONP distribution in the hearts. The center column is μCT images of heart cross sections in HU 0–500, where the VS55 could be distinguished from EC and the residue of sIONPs could be detected in this HU region. The right column is the histograms of the μCT from all cross sections for each case. The *x*-axis is HU, and the *y*-axis is the percentage of total pixels. The signal from EC, VS55, and sIONP could be distinguished as shown in the histograms. The μCT data showed left ventricle distension in the type A heart. The type B heart showed better washout comparing to the type A heart.

When sIONPs were loaded into the heart, the heart turns uniformly black regardless of the cannulation type due to iron accumulation (Figure 1a). To ensure that the hearts were saturated with sIONPs, the Fe concentrations in the effluents from hearts cannulated by the type A and type B methods were compared to the Fe concentration in the loading solution. The effluents from the hearts perfused sIONP/VS55 reached equilibrium Fe concentration after perfusion with 4 mL of perfusate volume (Figure 1c). Therefore, 4 mL of sIONP solution was used thereafter. The wet weight (*p* < 0.01) and Fe concentration/ dry weight of sIONP-loaded type A hearts (*p* < 0.001) were significantly greater than those of type B hearts (Figure S4, Supporting Information, and Figure 1d). The higher sIONP content in the type A hearts presumably arose from distension of the chambers, as shown by μCT imaging (Figure 2). The distensions of the LV and LA can be observed in the μCT images of a sIONP-loaded type A heart. To quantify these distensions, we analyzed the μCT images by creating a histogram of 50 axial cross sections across type A and type B hearts. We interpreted the regions with higher radiodensity (measured in Hounsfield units [HU] and averaging ≈750 HU) as chambers having an increased sIONP concentration, whereas the regions with lower HU (average ≈450 HU) represent the sIONP-loaded myocardium (Figure S5, Supporting Information). This interpretation yielded a chamber-to-myocardium volume ratio of 43/57 in type A versus 13/87 in type B. We interpret this as significant chamber distension (43% vs 13% of total volume) in type A hearts versus type B hearts (Figure S6, Supporting Information) and therefore selected the type B method over type A, as distension is known to depress LV performance.^[26a]^

Our previous studies have shown that the sIONPs were seldom taken up by the cells due to their polymeric (i.e., PEG) surface coating.^[16b]^ Furthermore, perfusion was carried out at a low temperature, which would inhibit active transport mechanisms of endothelial cells, tissue macrophage, or other cell populations that might come into contact with the sIONPs. For this reason, we expect the sIONPs to remain primarily in the extracellular luminal (vascular and heart chamber) spaces, thereby resulting in minimal Fe residue post nanowarming and washout. Our μCT imaging has a resolution of 17 μm, which allows the visualization of larger sIONP filled vasculature, although it cannot resolve at the capillary level and therefore cannot formally demonstrate if sIONP remain exclusively in the vasculature. To assess the further possibility of residual iron outside of the vasculature, tissues from sIONP-loaded hearts and sIONP-washed-out hearts were processed for histology with Prussian blue staining to detect residual iron. The images from these tissues showed blue staining (sIONPs) in the vasculature, and no sIONPs in the parenchymal spaces of sIONP loaded hearts (Figure S7, Supporting Information) suggesting sIONP remain solely in the vasculature.

To define the Fe concentrations in the heart muscle (inclusive of the vasculature), we selectively flushed the sIONPs from the heart chambers. CT and magnetic resonance imaging (MRI) were used to verify the removal of sIONPs from the chambers of the heart. The μCT images showed that the sIONP density in the muscle remained comparable to that in the fully sIONP-loaded hearts (Figure S8a, Supporting Information). Sweep imaging with Fourier transformation (SWIFT) MRI is able to measure Fe concentrations between the line broadening limit of detection for conventional MRI and the lower limit of detection of μCT.^[30]^ Although SWIFT-MRI R_1_ maps could not be acquired with the fully loaded chambers due to extreme line broadening exceeding practically available encoding bandwidth,^[30b]^ it was possible to acquire R_1_ maps once the chambers were unloaded. The decrease of R_1_ values in the chamber (3–4 1/s) compared to the muscle (7–9 1/s) indicated a decrease of sIONP concentration within the chamber (Figure S8b, Supporting Information). In addition to this imaging data, we were able to directly quantify Fe in the heart muscle by performing ICP-OES and found the Fe concentration in the heart muscle to be 1.47 ± 0.36 mg Fe mL^−1^ after loading.

#### 2.1.3. sIONP Washout

Although the sIONPs cause little cellular toxicity even at extremely high concentration (10 mg Fe mL^−1^),^[16b]^ the removal of loaded sIONPs is clearly important for the eventual clinical use of these hearts. Photographs of type A and type B hearts are shown pre-sIONP loading, post-sIONP loading and postwashout (Figure 1a). Visually, the washout efficiency of the type B method was superior, as clear patches of residual sIONPs could be observed in the ventricles of some type A hearts and not in any type B hearts. The superior Fe washout of the type B heart was further quantified by ICP-OES (Figure 1d, *p* < 0.05), and imaged by T2*-weighted MRI (Figure 1e) and μCT (Figure 2). The ICP-OES data showed that 93% of the loaded sIONPs were removed during unloading. The residual Fe in the washed-out heart (8 × 10^−4^ mg Fe mg^−1^ heart dry weight) was lower than a known tolerable Fe concentration in vivo in a mouse kidney 24 h post-i.v. injection with a similar type of sIONPs (2.4 × 10^−3^ mg Fe mg^−1^ kidney dry weight).^[16b]^ Residual sIONPs were visible in the unloaded hearts within the T2*-weighted MRI producing a hypointensity artifact due to the high Fe concentration. Previous work had demonstrated that the upper limit of quantification for sIONPs through T2* images acquired with similar parameters, in 1% agarose was 10.1 μg mL^−1^.^[30b]^ Furthermore, the washed-out type A heart showed a higher average radiodensity (148 HU) than the washed-out type B heart (121 HU) by μCT. A general relationship between IONP and increased HU is shown in Figure S5 (Supporting Information). Based on the superior washout efficiency of the type B method, we continued with only this approach in subsequent studies.

### 2.2. VS55 Loading and Removal via the Current Perfusion Method

#### 2.2.1. VS55 Loading in Rat Hearts

The VS55 loading efficiency was assessed by μCT. Heart tissues saturated with known concentrations of VS55 were first imaged by μCT to calibrate the μCT signal to the VS55 concentration (Figure S9, Supporting Information). Note, the μCT settings for VS55 calibration and VS55 loading efficiency measurements are different from that for sIONP detection (described further in the Experimental Section). Relative VS55 density was calculated from the calibrated μCT images and the resulting distribution of VS55 in the heart is shown in Figure S9 (Supporting Information). The chambers showed the highest VS55 concentration (≈100%), and the LV myocardium showed the lowest VS55 concentration (≈80%). Taken together, the average whole heart VS55 concentration was ≈86%. Although the tissue was not 100% equilibrated, Peyridieu et al. has reported that the critical rates (CCR, CWR) in CPA-loaded tissues can be dramatically less than those required for CPA containing solutions alone.^[31]^ For example, the CWR of 30% 2,3-butanediol CPA was 400-fold decrease in kidney tissue than in CPA solution alone.

#### 2.2.2. VS55 Removal from Rat Hearts

VS55 was removed from the hearts by performing the loading steps in reverse order (Figure 1b). Both VS55-loaded and unloaded hearts were imaged by μCT and compared with control hearts perfused with CPA carrier (Euro-Collins) alone (Figure 2). The μCT showed that the control and unloaded hearts were clearly different than the loaded heart. To quantify these differences, we mapped a histogram of the HU across the hearts and found that the HU distribution in the control heart (average HU = 96) was different from that in the VS55-loaded heart (average HU = 409) and very close to that in the VS55-unloaded heart (average HU = 102). These data confirm our ability to load, and subsequently unload, CPA in the heart chambers and myocardium using our perfusion protocol.

### 2.3. Cooling and Rewarming of Rat Hearts

#### 2.3.1. Cooling of Hearts

**Figure 3.**
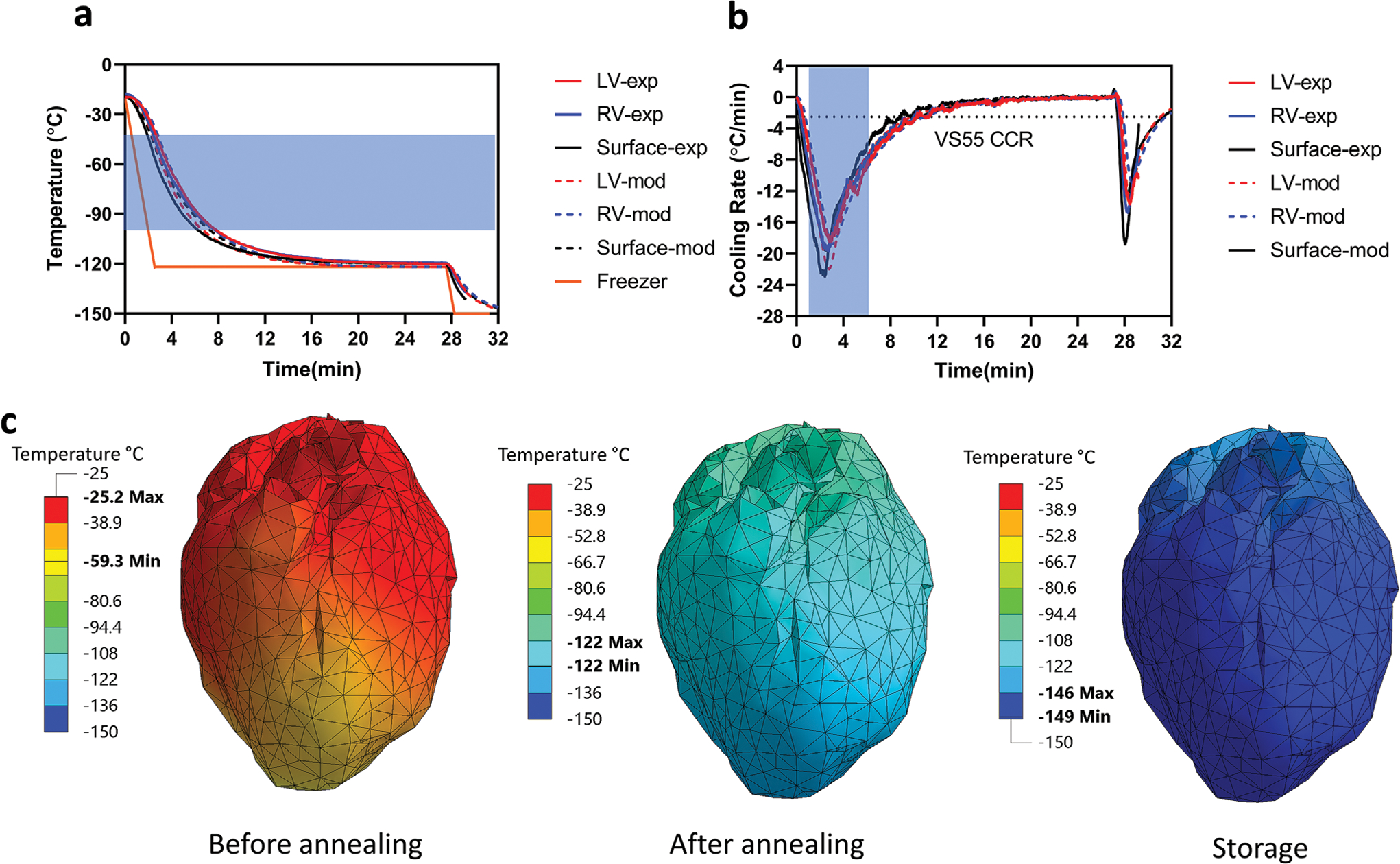
Experimental and modeling data for successfully cooled hearts. a) Measured thermal history by the three probes placed during experimentation, compared with computer simulation results at the same locations (i.e., modeling). The control rate freezer temperature was held at −122 °C (1 °C higher than the *T*_g_) for 25 min as the annealing step (orange line in the plot), when the temperature throughout the heart equilibrated before reaching *T*_g_. b) Experimental and calculated cooling rates for the probes presented in (a). The blue region indicates the temperature region for particular danger from ice growth (−100 to −40 °C). c) Color map representing the temperature distribution of a heart during cooling. From left to right: the cooling chamber reached the temperature of −122 °C (before annealing), after thermal equilibration at −122 °C (after annealing), and close to equilibrium around −150 °C (storage).

The sIONP/VS55-loaded heart (≤2 mL) was placed in a 2 × 3-in. bag surrounded by 10 mL of 3.75 mg Fe mL^−1^ sIONPs in VS55. The sIONP concentration in the surrounding solution was chosen to match the heart heating rate. A schematic diagram of the heart, bag, and cooling apparatus is shown in Figure S10 (Supporting Information). To record the temperature, three fiber optic temperature probes were placed in or on the heart: one in the LV, one in the RV, and one glued on the surface of the heart (Figure S10, Supporting Information). The locations of the fiber optic probes were verified by μCT (Figure S10, Supporting Information). The sample was placed in a controlled rate freezer, and a cooling protocol was applied as shown in Figure 3a. An annealing step at 1 °C above the VS55 glass transition temperature (*T*_g_) was added to the cooling protocol to reduce mechanical stress buildup prior to entering the glassy state.^[32]^

To support the experimental thermometry observations, we also performed finite element modeling to estimate the thermal behavior not only at the sites where the thermocouples were placed, but throughout the heart. Figure 3a displays the measured thermal history of the three temperature probes used for experimentation, as well as computer simulation results at the same locations (i.e., modeling). The experimental data and modeling results are in good agreement, which validates the computational model and framework. Figure 3b displays the cooling rate histories at the three probe locations. Ice growth requires water diffusion to the ice crystal and therefore cannot occur below the glass transition temperature (*T*_g_). Indeed, the peak ice growth rate is about 10 °C below the melting point (*T*_m_).^[33]^ For VS55, the temperature range considered hazardous for ice crystal formation and growth is generally considered to be between −100 and −40 °C.^[12]^ The blue area marked in the figure highlights the temperature range where the system is at significant risk of crystal growth within the time scale of cooling. As can be seen from Figure 3b, the cooling rate history generally exceeds the critical cooling required to avoid crystallization (marked with dotted line). Figure 3c displays the temperature distribution superimposed on the surface of the heart model at selected points in time, while the minimum and maximum values within the domain are highlighted on the temperature scales. As can be seen from Figure 3c, the temperature distribution in the heart muscle is in the range of −59.3 to −25.2 °C, when the cooling chamber reached the temperature of −122 °C (*T*_g_) for the first time. The significant temperature difference between the heart and the cooling chamber is primarily affected by the thermal mass of the CPA and specimen, as well as the cooling rate in the cooling chamber. It may be possible to alter the temperature distribution within the heart by changing its orientation within the bag or by changing the volume of the CPA surrounding the heart. Adding an annealing step to the protocol, just above the glass transition temperature (i.e., a temperature hold at −122 °C, near *T*_g_ [−123 °C for VS55]) is aimed at reducing thermomechanical stress and, thereby, reducing the likelihood of structural damage.^[14a,34]^

**Figure 4.**
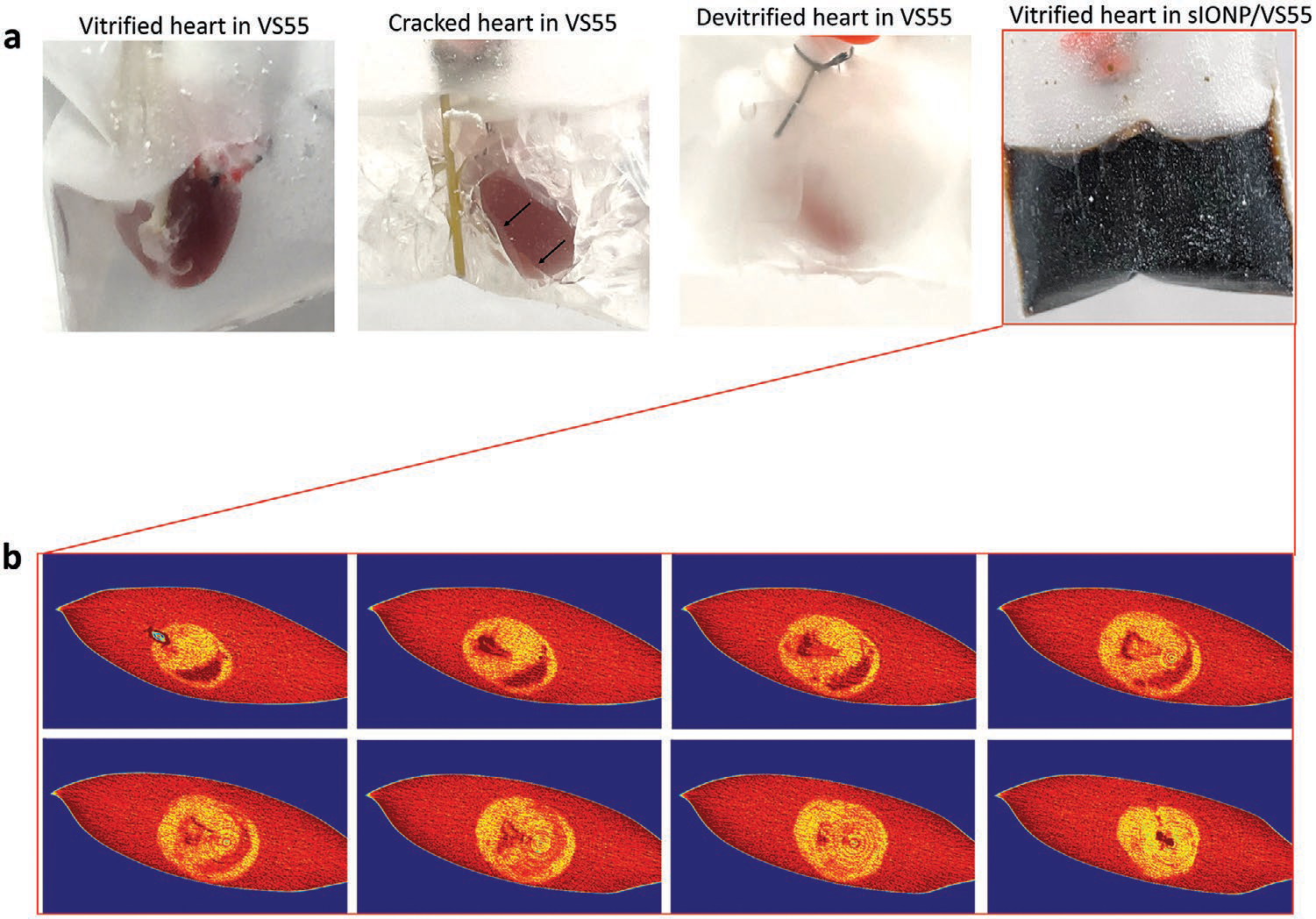
Gross and μCT images of success and failure of cooled hearts. a) Photos of vitrified (transparent), cracked (arrows show the cracks) and devitrified (white indicates ice formation) hearts in VS55 and a vitrified heart in sIONP/VS55. b) μCT images of a sIONP/VS55 loaded heart vitrified in sIONP/VS55. No crack or ice crystal was observed in the μCT cross sections of the vitrified heart after cooling in sIONP/VS55. The cross-sections are taken from bottom to top, and the displacement between the two cross-sections is 1.33 mm in the z-direction.

Figure 4a shows photos of vitrified, cracked, and devitrified (crystallized) hearts in VS55. Cryobags containing vitrified hearts in CPA solution are clear with hearts appearing the same as hearts suspended in CPA at room temperature. In contrast cracks and ice formation can be easily identified in cases with insufficient cooling or excess thermal stress during cooling (failure conditions). A photo of a successfully vitrified heart in sIONP/VS55 is also shown in Figure 4. The exterior texture looks uniform without visible cracking or ice formation, but due to the opacity of sIONP solutions, we are unable to visually examine the state of the interior of the bag and of the heart. Thus, we used μCT to look inside the sample. Figure 4b shows cross sections of a VS55/sIONP loaded and cooled heart within the bag. The μCT images show a uniform signal without visible fractures or ice crystal formation suggesting vitrification. Ice formation will cause an uneven and lower HU throughout the sample which appears granular, as shown in our previously published work.^[13]^ Example μCT images of a cracked heart are shown in Figure S11 (Supporting Information). The resolution of cryo-μCT is 61 μm (with instrument settings optimized for detecting cracks), which is sufficient to detect large cracks but may still miss microcracks and micro-ice formation. Note that this resolution is lower than the room temperature measurement shown in Figure 2.

#### 2.3.2. Rewarming of Vitrified Hearts

**Figure 5.**
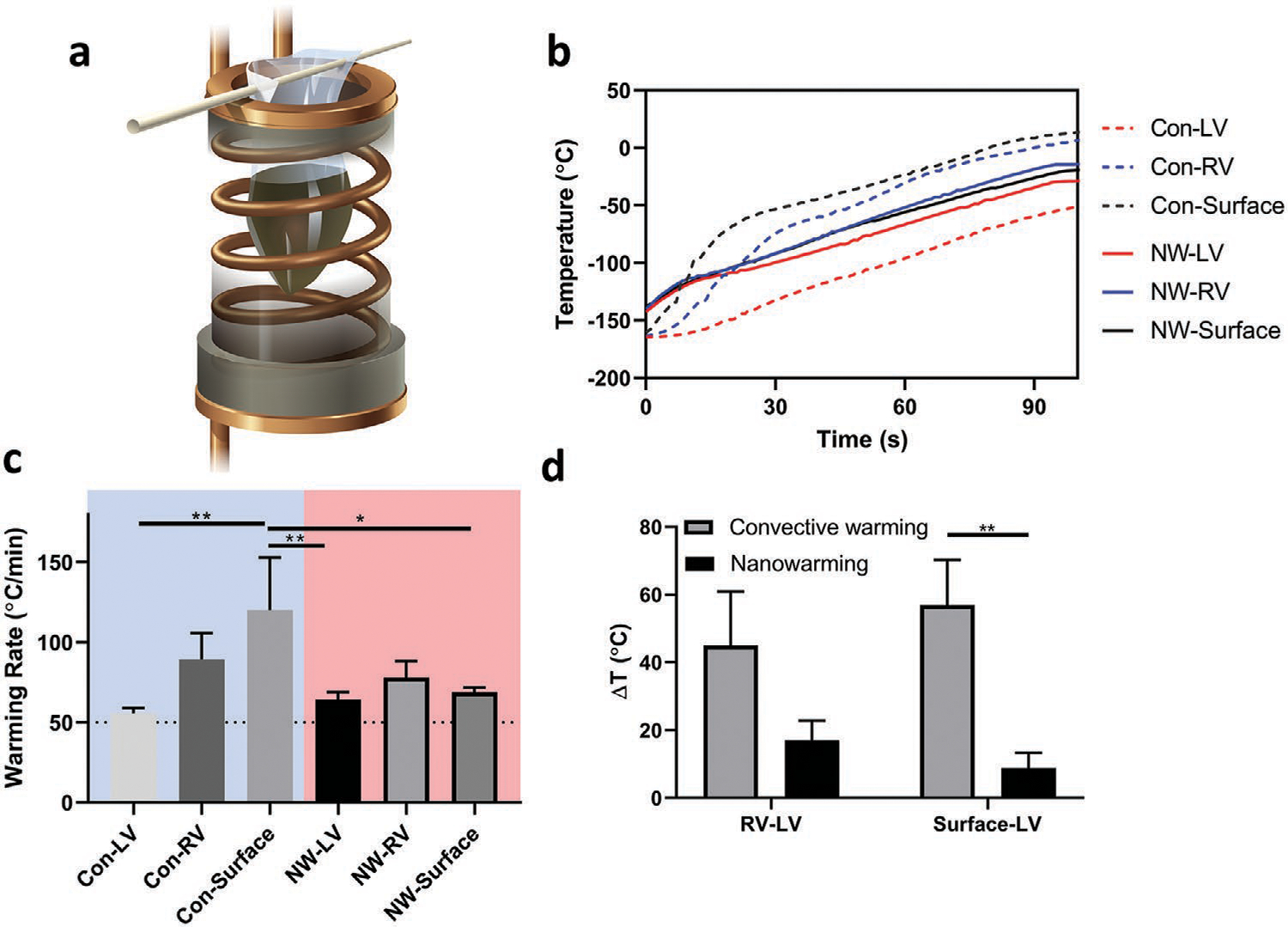
Experimental setup and data showing fast and uniform warming rates with nanowarming in comparison to convective warming. a) Illustration of a sample rewarmed in a RF coil. b) Temperature profiles of representative convectively warmed and nanowarmed hearts. The temperature difference which drives thermal stress was larger in convectively rewarmed heart than the nanowarmed heart. c) Warming rate in convectively warmed and nanowarmed hearts. *n* = 3. d) Maximum temperature difference between the fiber optic probes during rewarming in convectively warmed hearts and nanowarmed hearts. *n* = 3. Legends: Con-LV (convectively cooled left ventricle), Con-RV (convectively cooled right ventricle), Con-surface (convectively cooled heart surface). NW-LV (nanowarmed left ventricle), NW-RV (nanowarmed right ventricle), and NW Surface (nanowarmed heart surface).

The most common way to rewarm a vitrified sample is by convective warming. To test this approach we use immersion in a 37 °C water bath to achieve the most rapid rate possible.^[15,35]^ While convective warming is adequate to rewarm small volumes (<3 mL), in larger samples it is unable to rewarm with sufficient speed and/or uniformity to avoid ice formation (devitrification) or fractures.^[14c]^ Herein, we compare the physical characteristics of convectively warmed and nanowarmed vitrified hearts. To assess convective warming, VS55 loaded hearts were placed in the same bags and same volume of surrounding VS55 as the nanowarmed heart, but without sIONPs, and cooled to a vitrified state according to the same cooling protocol. Convective rewarming was conducted in a water bath set to 37 °C, and nanowarming was performed in a 15 kW RF coil at 64 kA m^−1^ and 185 kHz (Figure 5a). Temperatures were recorded in the same three locations described above. Representative warming profiles are shown in Figure 5b. Due to the flat shape of the bag and the small size of the rat heart, both convective warming and nanowarming showed warming rates faster than the VS55 CWR (Figure 5c). However, the convective rewarming profile showed larger temperature gradients due to differential heating on the surface versus interior of the hearts (Figure 5d). On the contrary, nanowarming initiated by the sIONPs induced relatively uniform warming throughout the sample. Of note, alternative methods to convectively rewarm in a more uniform way such as noted in Fahy et al.^[36]^ are much slower and run the risk of ice crystallization. In essence, convection is able to achieve rapid or uniform rates, but not both simultaneously, as shown here for nanowarming.

**Figure 6.**
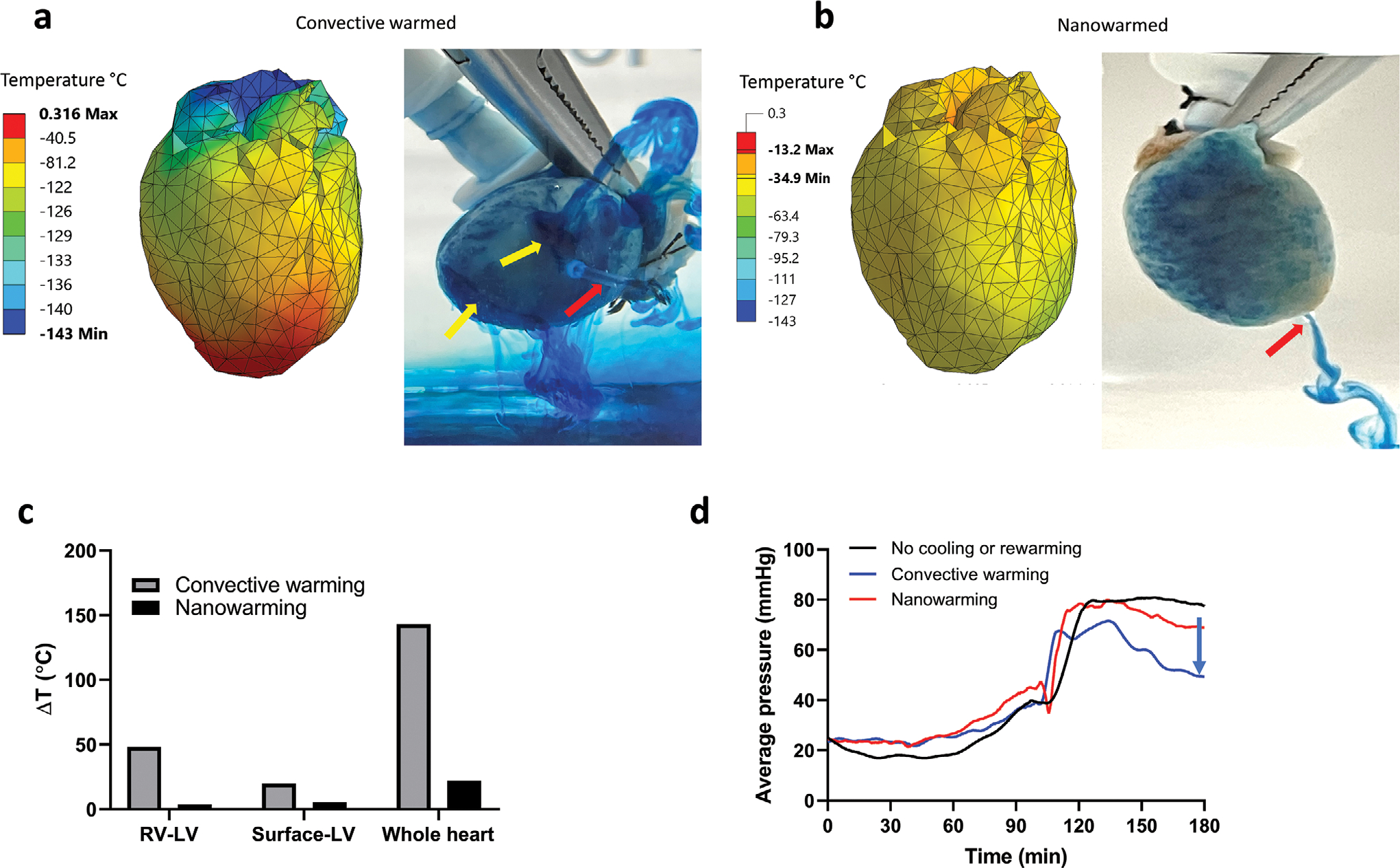
Computational modeling supports nanowarming success and convective failure during rewarming. a) Computer simulation results of a convectively rewarmed heart. The heart was shown to have cracks as indicated by the yellow arrows identifying sources of leaking methylene blue in addition to the catheter drainage (red arrow) during Type B perfusion. b) Computer simulation results of a nanowarmed heart. The photo shows an intact heart during methylene blue with a red arrow identifying the catheter in the left ventricle during Type B perfusion. c) Maximum temperature differences between right ventricle (RV), LV (left ventricle), surface-LV, and across the whole heart predicted by modeling. d) Comparison of average perfusion resistance pressure in a control heart (no cooling or warming), convectively warmed, and nanowarmed hearts. The convectively rewarmed heart showed lower perfusion pressure indicated by the blue arrow and likely indicating vascular leakage due to cracks.

The experimental measurements of temperature are limited to discrete positions; therefore, we complemented this with further modeling to characterize the temperature throughout the whole heart during rewarming. Figure 6a,b and Figure S12 (Supporting Information) display the temperature distribution on the heart muscle surface at the end of two rewarming protocols: convective heating and nanowarming, respectively. It can be observed from Figure 6a that for convective rewarming the minimum temperature in the heart is −143 °C when the first portion of the heart approaches 0 °C. This difference is evidence of steep temperature gradients that can give rise to significant thermomechanical stress, which is the driving mechanism for the observed fractures.^[37]^ It can be observed from Figure 6b that the temperature variations in the nanowarmed hearts were much smaller, with a gradient of 22 °C when the points with the lowest temperatures surpasses −35 °C. These latter temperature values were above the heterogeneous nucleation point of VS55, suggesting no further risk for rewarming-phase crystallization. Figure 6c displays the maximum temperature difference between nanowarming and convective rewarming at the respective locations of the temperature probes.

To assess the impact of our warming methods on the hearts, we re-perfused the hearts and assessed both flow and pressure during CPA and sIONP unloading. Figure 6d shows the perfusion pressure of a convectively warmed heart, a nanowarmed heart, and a heart that was not subjected to cooling or rewarming. The pressure profiles (i.e., resistance measurements) of the nanowarmed heart and the heart not subjected to cooling and warming were comparable (*p* = 0.74), while the convectively warmed heart showed a lower perfusion pressure during the removal steps (*p* < 0.0001). As discussed above, the greater temperature variation in the convectively warmed heart could result in cracking. We assume that the pressure reduction observed after convective warming is due to compromise of the vascular system caused by such cracking. To demonstrate this possibility more clearly, we added methylene blue to the perfusate for both convectively warmed and nanowarmed hearts. Clamping the PA during perfusion resulted in clear leakage of methylene blue from the cracks on the surface of the convectively rewarmed heart, while the nanowarmed heart was intact and methylene blue was only observed to drain from the LV catheter (Figure 6).

After nanowarming, the sIONPs and VS55 were removed by the same removal procedure as in Figure 1b. A photo of the washed-out heart after nanowarming is shown in Figure S13 (Supporting Information). The residual Fe (1.2 × 10^−3^ mg Fe mg^−1^ dry weight) from the washed-out heart after nanowarming was not significantly different from that in hearts that were not subjected to vitrification and nanowarming (*p* = 0.062). The μCT image of the washed-out heart after nanowarming was also similar to that of the heart that was not subjected to vitrification and nanowarming (Figure 2). In summary, adding vitrification and nanowarming did not change the perfusion pressure or any of our other metrics of sIONP/VS55 washout.

#### 2.3.3. Heart Tissue Architecture Following Whole Organ Vitrification and Rewarming

**Figure 7.**
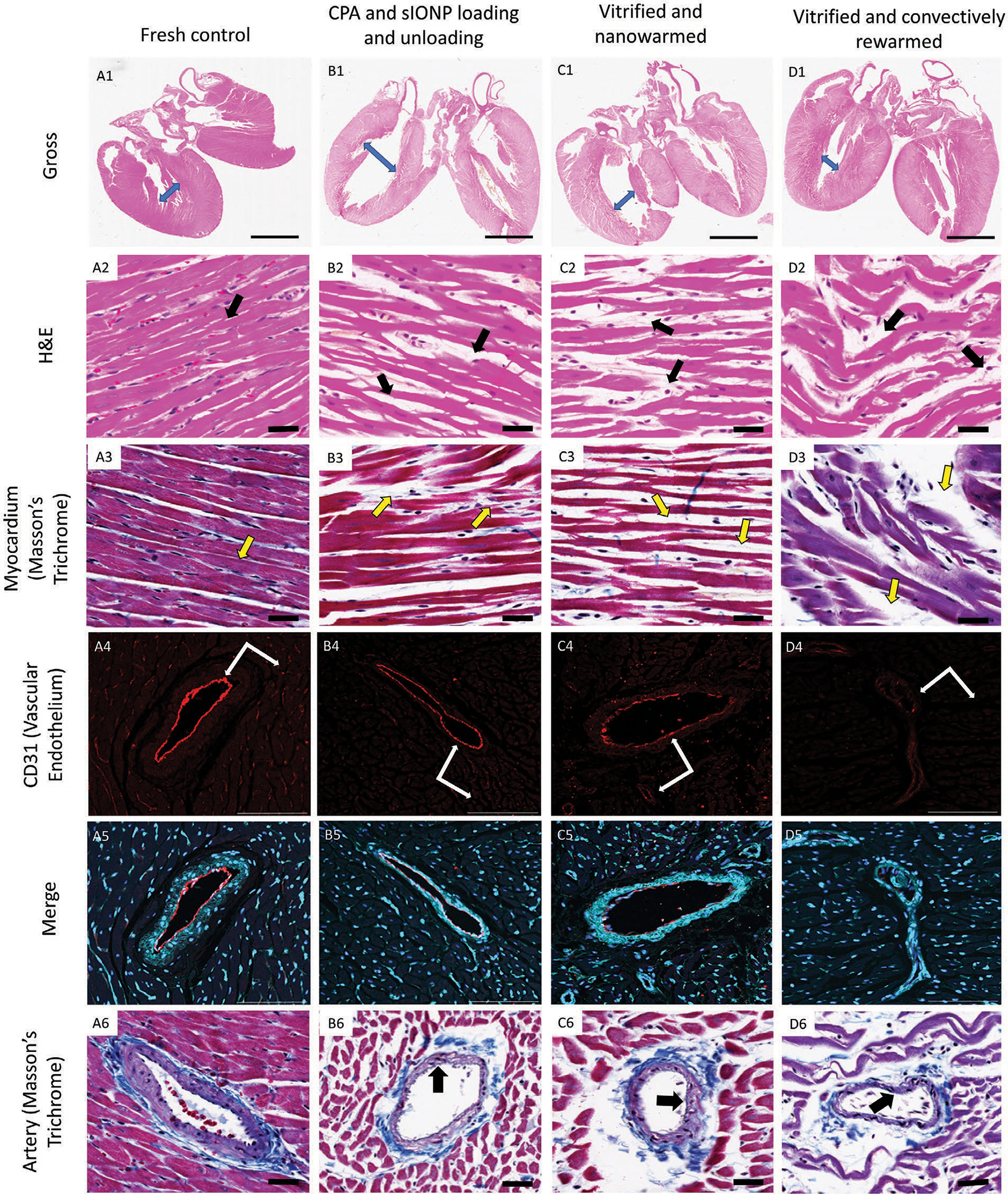
Histology and confocal imaging characterization. A1–D1) Gross histology of the heart on H&E comparing wall thickness and chamber volumes. Length of arrows represents the dilatation of chambers in comparison to control heart. A2–D2, A3–D3) Cardiac muscle stained with H&E and Masson’s trichrome. Arrows represent the separation of muscle fibers in all treatment groups. Conventional cryopreserved hearts represent extensive muscle disruption suggesting significant damage to tubular myofibrils and sarcomeres inhibiting intercalated disks transmitting electrical action potential between sarcomeres in contrast to vitrified-nanowarmed hearts (33.6×). A4–D4) CD31 labeled arterial endothelium in the left ventricle using fluorescence confocal microscopy. Arrows represent the integrity of the vascular endothelium in large arteries and intact smaller vasculature in the heart. Significant disruption of endothelial integrity in conventional cryopreserved hearts (20×). A5–D5) Merged confocal microscopy labeling vascular endothelium (CD31: red), galactose-*α*−1,3-galactose (cyan) representing carbohydrates (glycans) over the surface of muscles and blood vessels giving an architectural background and DAPI (blue) representing nuclear morphology (20×). A6–D6) Large arterial branches in the left ventricle stained with Masson’s trichrome (muscle: red; nuclei: deep blue; collagen: blue). Arrows representing arterial collagen matrix thickness alterations across treatment groups (33.6×).

Figure 7 demonstrates the morphologic and histologic alterations between the treatment groups and compares fresh (Column A), sIONP/VS55 loaded and unloaded (Column B), vitrified and nanowarmed (Column C), and convectively rewarmed (Column D) hearts. Low power/gross examination of hematoxylin and eosin (H&E) stained hearts with four-chamber bookended bisection (Row A) demonstrates larger chamber volumes in the ventricles of hearts that were perfused ex vivo (B1-D1) in comparison to fresh and non-perfused hearts (A1), although the gross morphology of each of the treatment groups appears comparable.

H&E staining of the left ventricular myocardium can be better appreciated at 20× magnification demonstrating more prominent separation of muscle fibers in both the treatment groups (B2 and C2) compared to the control (A2) with wellmaintained branching striated muscle fibers with intercalated discs and normal nuclear appearance. In contrast, the convectively warmed heart (D2) demonstrated significant morphological changes presumably due to ice formation during cooling and rewarming. The convectively rewarmed cardiomyocytes were wavy in appearance with disruption of some of the intercalated disc junctions between branching cells.

Masson’s trichrome staining of the (20×) cardiomyocytes of the left ventricular tissue demonstrated no significant morphologic changes between vitrified nanowarmed (C3) and nonvitrified hearts (B3) but did show some decreases in staining intensities within the cardiomyocytes without evidence of fibrosis. However, convectively rewarmed hearts (D3) show clear evidence of significant architectural disruptions of the muscle fibers, which is likely related to cellular injury during ice formation.

The endothelial layer in CPA/sIONP loaded and unloaded as well as vitrified and nanowarmed hearts appears intact (see anti-CD31 immunofluorescence, A4–C4), but were absent in convectively rewarmed organs (D4). Although, it was observed that there were some expansions of the perivascular spaces for all but control hearts, detected with Trichrome staining. Further, the collagen matrix in the arterial walls stained using the Masson’s trichrome also shows compositional alterations with vitrification, nanowarming (C6) and perfusion (B6) compared to control (A6), i.e., with extensive damage to arterial walls and collagen matrixes around convectively rewarmed arteries (D6).

In summary, it appears that CPA/sIONP loading and unloading leads to small histologic changes in rat hearts, but allows for preservation of the overall architecture and vascular endothelium. Notably, no further injuries occurred with vitrification and nanowarming. These data suggest that the main limitations for whole-heart vitrification may be CPA toxicity and/or perfusion damage rather than vitrification and nanowarming induced injuries, and these observations suggest that improvements in CPA delivery/removal may in time overcome limitations for functional recoveries of preserved hearts for subsequent transplant. Indeed, our preliminary optical mapping experiments using voltage-sensitive dyes demonstrated the presence of electrical activity in nanowarmed hearts (*n* = 3), which to some extent resemble that in control hearts (*n* = 3). Figure S14 (Supporting Information) demonstrates that nanowarmed hearts (one out of three) can successfully respond to pacing, while their mean action potential duration is significantly larger (139.05 ± 2.12 ms) than that for control heart (72.61 ± 7.78 ms, *p* < 0.05), suggesting possible alterations in electrophysiological properties. Figure S14 (Supporting Information) also indicates that electrical activity can successfully propagate in nanowarmed hearts (see 2D AT naps), albeit in a compromised way. All three nanowarmed hearts showed the presence of arrhythmia (data not shown) during optical mapping experiments.

## 3. Conclusion

Herein, we report physical and biological demonstration of mammalian heart controlled machine perfusion of CPA and sIONPs, vitrification, nanowarming, and washout. Experimental measurements and modeling of the thermal response demonstrated that the nanowarmed hearts achieved rates sufficient to vitrify VS55 during cooling and avoid cracking and devitrification during warming. In contrast, experiments and modeling confirmed that convectively rewarmed hearts cracked due to inherent non-uniformity in the process. Further, nanowarmed hearts were shown to be largely equivalent in tissue integrity and morphology to sIONP and CPA loaded and unloaded hearts: they retained some electrical activity, and were clearly superior to convective controls. This study demonstrates that a whole rat heart can be physically vitrified and rewarmed while retaining tissue integrity, morphology, and some electrical connectivity over convective failures, thereby establishing an important milestone in providing viable and intact nanowarmed hearts for future transplants or other biomedical use and applications.

## 4. Experimental Section

### Chemicals:

Tetraethylorthosilicate (TEOS), polyvinylpyrrolidone (PVP10, average molecular weight 10 000), and chlorotrimethylsilane (TMS, >99%) were purchased from Sigma Aldrich. 2-[Methoxy(polyethyleneoxy)-propyl]9–12-trimethoxysilane (PEG-silane) was obtained from Gelest, Inc. Ethanol (99%) was purchased from Pharmco-Aaaper. EMG308 ferrofluid was purchased from Ferrotec. Ammonium hydroxide (NH_4_OH, 28%) was obtained from Avantor Performance Materials.

### sIONP Synthesis and Characterization:

The details of sIONP synthesis were published in a previous work.^[24]^ In brief, 48 g PVP10 was dissolved in water, and 1.440 g Fe EMG308 (Ferrotec) was added to pre-probesonicated PVP10 solution (total water volume is 432 mL) and subsequently probe sonicated (Q500, Qsonica) for 45 min. Then, the mixture was added to 3.2 L ethanol and probe sonicated for another 45 min while stirring. Next, 160 mL ammonia was added to the 4 L reaction vessel (LG-8082–104, Wilmad-LabGlass) while stirring with an overhead mechanical stirrer (OS20-S Waverly), and 60 mL of TEOS was added afterwards while stirring. PEG silane (15 mL) was added to the mixture after 1 h, and stirring was continued. TMS (2.25 mL) was added after another half an hour. After the reaction, the reaction solution was concentrated by a rotary evaporator, and the sIONPs were collected and purified via repeat centrifugation. Each batch of sIONPs was characterized by DLS (Brookhaven Zeta PALS instrument), TEM (Tecnai T12, FEI, OR), and ICP-OES (Thermo Scientific iCAP 6500 dual-view ICP-OES).

### Rat Heart Procurement and Cannulation:

Male Lewis and Sprague-Dawley rats, 2.5–3.5 months old, weighing 250–350 g, were used as a heart donor. The protocol (1905–37029A) was approved by the Institutional Animal Care and Use Committee (IACUC) at the University of Minnesota. Rats were anesthetized with 4% isoflurane and 1 L min^−1^ oxygen. A toe pinch test was performed to confirm the adequacy of anesthesia. The abdomen and thoracic area were shaved and disinfected with 70% ethanol.

### Type A Cannulation:

A transverse abdominal incision was made, and the chest was opened through the right and left axillary lines. 500 IU of heparin solution was injected intravenously. After 1–2 min, the suprahepatic vena cava (SHVC) was clamped using a bulldog clamp. The heart was flushed with 20 mL of UW solution mixed with 500 IU of heparin through the SHVC. Once the flush was done, the SHVC and SVC were ligated. The ascending aorta and pulmonary artery were cut, and the pulmonary veins were ligated. The heart was explanted and cannulated in the ascending aorta. A mixed solution of 10 mL of UW and 250 IU heparin was injected into the cannula.

### Type B Cannulation:

This cannulation approach was performed in the same manner as the above-written method for type A cannulation, but following that procedure an 18G needle was used to puncture the LV and a 20G, 5–8 mm long bulb tip catheter (FTP-20–30, Instech laboratories) was inserted into the LV. Afterward, 5 mL of UW and 125 IU of heparin were injected through the cannula inside the ascending aorta and the heart was placed in a container of cold UW solution.

Other tested cannulation methods include the following: Type A cannulation with 20G bulb tip catheter insertion into the LV via the left atrial appendage puncture, and after successful completion of type A cannulation, a small defect was created on the left atrial appendage and the mitral valve of the heart was removed using a 3 mm aortic punch (Medtronic, USA).

### VS55 and sIONP Loading and Removal in Rat Hearts:

The hearts were submerged in an organ bath at 4 °C. The perfusion of VS55 was done step-wise (Euro-Colins, 18.7%, 25%, 50%, 75%, 100%, 15 min in each step) at a constant flow rate (1 mL g^−1^ min^−1^). 10 mg Fe mL^−1^ sIONPs in VS55 was perfused into the heart after VS55 loading at 1 mg mL^−1^. The removal protocol was opposite of the loading protocol. The perfusion pressure (resistance) and temperature in the organ bath were recorded during perfusion.

### μCT Imaging:

Samples were scanned in a μCT imaging system (NIKON XT H 225, Nikon Metrology, MI). 3 μCT parameter settings were used to achieve different imaging goals: detect the CPA distribution inside the heart tissue, detect the IONP distribution inside the heart chamber and capillaries, and detect vitrification. The optimized parameters were adopted from previous work to study the CPA distribution inside the heart after step-wise loading.^[38]^ The accelerating voltage was set to 65 kV, and the current was set to 95 μA. The resolution was 0.029 mm. The same voltage and current were used to detect vitrification of the heart, and to fit the low-temperature setup into the scanning VOI, the samples were moved slightly away from the X-ray source; therefore, the resolution decreased to 0.061 mm. Moreover, the voltage (121 kV) and current (150 μA) were increased to detect the presence of the metal component contained in the IONPs.^[39]^ Meanwhile, a 1-mm aluminum filter was placed between the source and the object to reduce the beam hardening effect.^[40]^ The resolution was also increased to 0.017 mm to obtain more detailed information inside the chamber and capillaries.

X-ray attenuation is represented in HU—a clinical measurement that normalizes X-ray attenuation values by the difference between those of water and air at 20 °C.^[17,41]^ For the vitrification setup, the water and air samples for HU calibration were insulated with Styrofoam and placed in chamber A (Figure S15, Supporting Information) at room temperature, while the cryobag (Item No. 1959T11, McMaster-Carr., Robbinsville, NJ) containing the vitrified heart was kept at LN_2_ vapor temperature (−150 °C). A typical scan took 30 min to finish, and the temperature history monitored in chamber B showed that during the 30 min period, the temperature changed from −174 to −158 °C, both of which are below the glass transition temperature; therefore, the sample was kept in the vitrified state.

μCT images were reconstructed with pre-processing software (3D CT pro, Nikon Metrology, MI) to reduce the beam hardening effect and improve the image quality. The images were then imported as unsigned 16-bit float images, post-processed (VGstudio Max 3.2, Volume Graphics, NC), and exported as DICOM images for a final analysis using MATLAB (MathWorks). The grayscale values were transferred into HU based on the air and water samples in MATLAB.

### MRI Imaging:

All MR images were acquired in a 9.4 T, 31 cm bore magnet (Magnex Scientific, Yarnton, UK) interfaced to a research console (Agilent Technologies, Inc., Santa Clara, CA) with volume transmit/receive coil with an inner diameter of 3 cm (Varian, Palo Alto, CA) for Gradient Echo (GRE) MRI and with a homemade transmit/ receive semi-volume coil consisting of tree loops of 2.2 cm diameters stocked with 3mm gaps and connected in parallel to improve the RF field homogeneity for SWIFT. Hearts were placed in a PET 50mL falcon tube on a Teflon holder for GRE MRI and in 2 cm diameter glass tube on a Teflon holder a surrounded by Euro Collins buffer for SWIFT MRI.

GRE acquisitions were used to produce 2D T2* images. Each image was acquired with bandwidth (BW) = 50 kHz, TR = 1.2 ms, TE = 3 ms, and acquisition time = 2.56 ms. Resolution = 391 × 391 μm^2^, slice thickness = 2 mm (FOV = 5 × 5 mm), angle = 20°.

The SWIFT 3D R1 weighted images were acquired using a Look-Locker method^[30a]^ with a multi-band (MB) SWIFT sequence^[42]^ for image readout using flip angle = 4°, acquisition delay = 2 μs, BW = 250 kHz, TR = 1.2 ms, gaps = 4, using 128 sidebands, voxel resolution = 250 × 250 × 250 μm^3^, and total acquisition time around 11 min.^[30a,42]^ The field-of-view (FOV) was 32 × 32 × 32 mm^3^ with image matrix size = 128 × 128 × 128 × 24 (x,y,z,t), number of grouped views (Nspiral) = 64 with number of views in each group (Nv) = 256, and 24 time points spaced linearly from 39.8 to 2185 ms. MB-SWIFT images were reconstructed using gridding and a fast iterative thresholding algorithm (FISTA) available at CMRRpack (http://www.cmrr.umn.edu/swift/). The R1 maps were produced voxel base with three-parameter Levenberg-Marquardt fitting and compensating the RF field inhomogeneity by method proposed by Deichmann et al.^[43]^

### Vitrification and Warming:

The hearts were vitrified in a controlled rate freezer (Kryo 560–16, Planer). The hearts were first equilibrated at −20 °C, and the freezer was set to cool to −122 °C at −40 °C min^−1^, isotherm at −122 °C min^−1^ for 25 min as the annealing step, cool to −150 °C, and isotherm at −150 °C > 5 min to achieve vitrification. Nanowarming was done in a 15 kW RF coil (AMF Life Systems LLC) at 92% power (≈62 kA m^−1^) and 185 kHz. Convective warming was done by forcing the vitrified bag into a 37–40 °C water bath. The temperature in the LV and RV and on the surface of the heart was recorded during cooling and rewarming.

### Heart Digestion:

The hearts were dried by either a lyophilizer (FreeZone 6, Labconco) or a vacuum oven (Thermo Scientific) depending on the presence of organic solution within the heart. The dried hearts were ground into powder, and ≈50 mg of the powder was predigested with 0.3 mL H_2_O_2_ and 0.6 mL HNO_3_ overnight in a 6 mL microwave digestion vessel (Savillex). The samples were sealed in the microwave digestion vessel inside a 60 mL microwave digestion vessel with 10 mL H_2_O in the larger vessel the next day. The microwave digestion procedure was performed in a domestic microwave with the following sequence: 50% power for 3 min, cool down, release pressure, 50% power for 3 min, cool down. The digested solution was then diluted to 10 mL with 2% nitric acid. The resulting solution was then digested similarly to the IONP samples in a solution of 0.3 m ascorbic acid and 0.3 m HCl at 60 °C for 3 h.

### Modeling:

As specimen-specific geometric model cannot be obtained without adversely affecting cryopreservation success, a generic model was used, scaled to meet experimental conditions (rat heart volume of 0.686 mL^[44]^). An MRI-based geometrical model of a heart was obtained from the Visible Heart Laboratory at the University of Minnesota.^[45]^ A model for the cryobag containing the heart was generated using Solidworks, as described previously,^[37]^ scaled to meet experimental conditions.

Heat transfer within the organ-CPA system is assumed to be governed by heat conduction

(1) $$C\dot{T}=\nabla \cdot (k\nabla T)+\dot{q}$$

where *C* is the volumetric specific heat, *T* is temperature, *k* is thermal conductivity, and *q* is the heat generated due to nanowarming. Continuity in temperature and heat flux is assumed on all internal boundaries between the subdomains in the system, and a convective boundary condition is assumed on the cryobag outer surface.

The heat transfer coefficient between the cryobag and the cooling chamber was evaluated using a least-square parametric estimation method, where the optimum heat transfer coefficient was selected such that the temperature difference between experimental data and simulated results is minimized

(2) $$F_{0}=\sum_{i=1}^{3}F_{i}=\sum_{i=1}^{3}\sqrt{\frac{1}{n}\sum_{p=1}^{n}{\left[T_{m.i}^{p}(x,y,z)-T_{s.i}^{p}(x,y,z)\right]}^{2}}$$

where *F*_0_ is the global target function, *F*_*i*_ is the target function at sensor location *i* (1 = left ventricle, 2 = right ventricle, 3 = surface sensor), *T*_*m*.*i*_ is the measured data at location *i*, *T*_*s*,*i*_ is the simulated temperature at the same location, *p* is the time index of measured data, and *n* is the size of the data set. Using this method, the best-fit heat transfer coefficient in this study is found to be 80 W m^−2^ K^−1^ during cooling.

To achieve rapid rewarming rate during the convective heating experiment, the cryobag was immersed in a well-stirred water bath, set to a constant temperature of 40 °C. While the specific heat transfer coefficient between the cryobag and the warm water remains unknown, it is orders of magnitude higher than that of free convection in air during the nanowarming experiment (≈15 W m^−2^ K^−1^). To estimate the highest possible rate of warming, a constant cryobag surface temperature (i.e., infinite convective coefficient) was assumed for computer simulations.

The volumetric heat generation during nanowarming was evaluated using measured SAR for VS55 mixed with silica-coated nanoparticles (sIONP = EMG-308 Ferrotec) excited at a field strength of 64 kA m^−1^ and frequency of 185 kHz

(3) $$\dot{q}=SAR\times C_{n}[W/L];\;SAR=\{\begin{array}{cc} 690.82W/gFe & T=-120^{{}^{\circ}}C\\ 415W/gFe & T=-20^{{}^{\circ}}C \end{array}$$

where *C*_n_ is the nanoparticles concentration in mg Fe/mL. Based on experimental data, the following nanoparticle concentrations were assumed for computer simulations: 10 mg Fe mL^−1^ in the heart chambers, 1.47 mg Fe mL^−1^ (based on ICP-OES) in the heart muscle, and 3.75 mg Fe mL^−1^ in the surrounding CPA within the cryobag. To simulate nanowarming of the cryobag at room temperature under free convection, a convective coefficient of 15 W m^−2^ K^−1^ was applied to the outer surfaces of the cryobag at 22 °C. SAR was approximated to be constant between the temperatures of −120 °C and −80 °C, having a value of 690.82 W g^−1^ Fe as measured in experiments and displayed in equation (3). Within the temperatures of −80 °C and −20 °C, SAR was assumed to be linearly decreasing from 690.82 to 415 W g^−1^ Fe, respectively. This approximation was based on experimental observations. For computational purposes, it was further assumed that the SAR remains constant even below −120 °C and that the decreasing linear slope of SAR between −80 °C and −20 °C continues up to 0 °C.

Consistent with previous studies,^[46]^ vitrified CPA properties were used instead of vitrified CPA-permeated heart. Thermophysical properties used for computer simulations are listed in Table S1 (Supporting Information). Simulations were performed using the finite elements analysis (FEA) commercial code ANSYS 19.1, using tetrahedral (SOLID87) elements. In total 31,180 elements and 1 s time steps were used to satisfy a mesh convergence analysis.^[47]^

### Histology:

Hearts were sectioned in the coronal plane to expose the four chambers, the aorta and the pulmonary trunk. The defected present in the LV apex represents the location of the catheter that was inserted to decompress the left ventricle during Langendorff type B perfusion (LV vent). Following each experiment, the heart was transferred to 10% neutral buffered formalin and paraffin embedded within 48 h. Using a microtome, 5 μm sections were prepared and stained with H&E, Masson’s trichrome (muscle: red; nucleus: blue; green/blue: collagen), and labeled with rabbit monoclonal recombinant anti-CD31 primary antibody (Abcam, Cambridge, UK) and fluorescent tagged with goat antirabbit IgG H&L (AF-647) preadsorbed secondary antibody (Abcam). The sections were also labeled with Isolectin Griffonia simplicifolia AF-488 conjugate (Thermo Fisher, Waltham, MA) which binds d-galactosyl residues of galactose *α*−1,3 galactose (Gal *α*−1,3 Gal). Nuclei were labeled with 4′,6-diamidina-2-phenylindole (DAPI).

### Confocal Microscopy:

Paraffin embedded 5 μm sections were deparaffinized using 4% paraformaldehyde solution and labeled with the recombinant Anti-CD31 antibody and conjugated with preabsorbed AF-647 secondary antibodies. Gal *α*−1,3 Gal labeling served as a background and outline vascular boundaries of arteries and veins. DAPI was used to label nuclei. Olympus Fluoview 3000 inverted confocal microscope (Olympus, Center Valley, PA) was used for imaging the sections.

### Optical Mapping Setup and Analysis:

Isolated ex vivo whole heart optical mapping experiments were performed as described previously.^[48]^ Rat control (*n* = 3), nanowarmed (*n* = 3), and electrically silent (*n* = 3) hearts were perfused using Langendorff perfusion setup with warm (37 ± 1 °C) Tyrode’s solution (in mm): NaCl 130, CaCl_2_ 1.8, KCl 4, MgCl_2_ 1.0, NaH_2_PO_4_ 1.2, NaHCO_3_ 24, glucose 5.5, and pH 7.4. After 15 min of stabilization, a bolus of the voltage-sensitive dye (di-4-ANEPPS, 5 μg mL^−1^) was injected into the hearts. Two green lasers (532 nm, Shanghai Dream Lasers Tech., Shanghai, China) were used to excite the hearts and the fluorescence signal intensity was recorded for using 14-bit, 80 × 80-pixel resolution cameras (Little Joe, RedShirt Imaging, SciMeasure) at 1000 frames per second.

The hearts were paced at basic cycle lengths (BCL) 180 and 200 ms for at least 40 stimuli to reach steady state, and optical movies corresponding to the last ten stimuli were recorded and analyzed. Optical APD was measured at 80% repolarization, and 2D APD and activation times (AT) (measured at 50% depolarization) maps were constructed to reveal the spatial distribution of APD and impulse propagation on the epicardial surface of the heart.

### Statistical Analysis:

Sample sizes are listed in the figure captions. In general, *n* = 3–7 for each statistical analysis. Statistical significance is indicated with asterisks: * *p* < 0.05; ** *p* < 0.01; *** *p* < 0.001; **** *p* < 0.0001. The error bars are standard deviations. The one-way and two-way analysis of variance (ANOVA) with Tukey’s multiple comparison tests (GraphPad Prism, GraphPad® Software, Inc.) was performed on data.

## Supplementary Material

supinfo

## Data Availability

The data that support the findings of this study are available from the corresponding author upon reasonable request.
