# Supplementary material for: Vitrification and Rewarming of Magnetic Nanoparticle-Loaded Rat Hearts
Source: Adv Mater Technol. Author manuscript; Available in PMC 2023 Mar 1. (PMC9164386; doi:10.1002/admt.202100873)
Supplement: supinfo [file NIHMS1745591-supplement-supinfo.pdf]

© 2021 Wiley-VCH GmbH

ADVANCED MATERIALS TECHNOLOGIES

Supporting Information

for *Adv. Mater. Technol.,* DOI: 10.1002/admt.202100873

Vitrification and Rewarming of Magnetic Nanoparticle-Loaded Rat Hearts

Zhe Gao, Baterdene Namsrai, Zonghu Han, Purva Joshi, Joseph Sushil Rao, Vasanth Ravikumar, Anirudh Sharma, Hattie L. Ring, Djaudat Idiyatullin, Elliott C. Magnuson, Paul A. Iaizzo, Elena G. Tolkacheva, Michael Garwood, Yoed Rabin, Michael Etheridge, Erik B. Finger, and John C. Bischof**

Supporting Information

Vitrification and rewarming of magnetic nanoparticle-loaded rat hearts

Zhe Gao†, Baterdene Namsrai†, Zonghu Han, Purva Joshi, Joseph Sushil Rao, Vasanth Ravikumar, Anirudh Sharma, Hattie L. Ring, Djaudat Idiyatullin, Elliott C. Magnuson, Paul A. Iaizzo, Elena G. Tolkacheva, Michael Garwood, Yoed Rabin, Erik B. Finger, John C. Bischof**

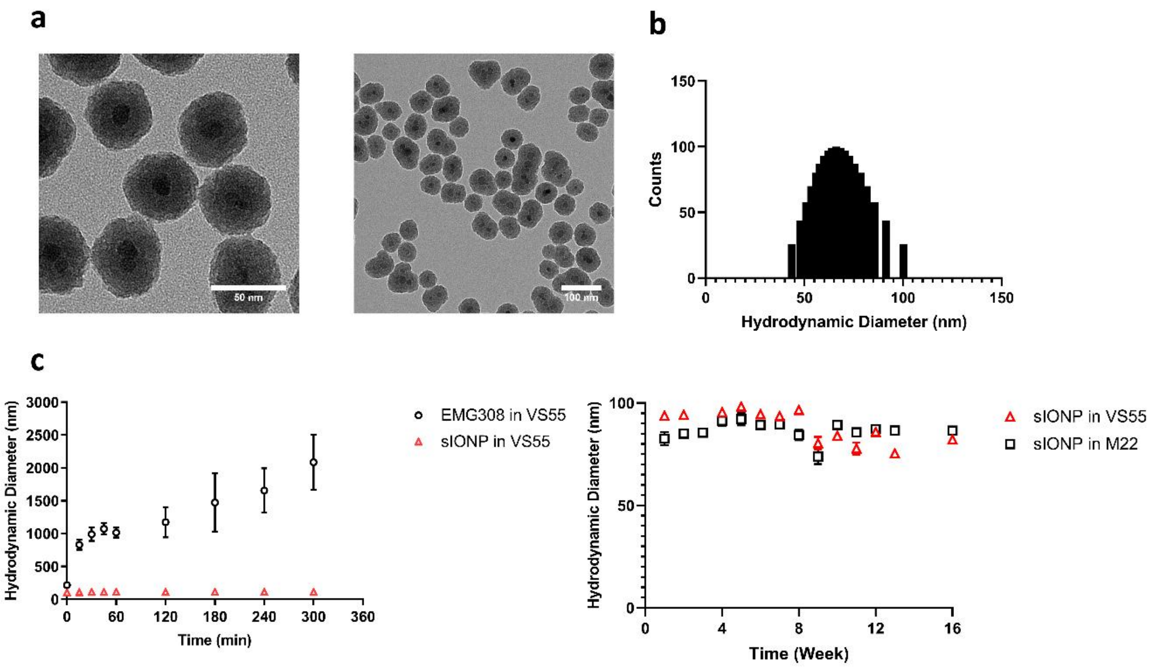

Figure S1. Characterization and stability in solution of silica-coated iron oxide nanoparticles (sIONP). A) Representative TEM images of sIONP, b) Hydrodynamic diameter distribution of sIONP, and c) EMG308 and sIONP hydrodynamic diameters in water and VS55 at low concentration (0.01g Fe/mL) over 6 hours (left) and sIONP hydrodynamic diameter in VS55 and M22 at 10 mg Fe/mL over 4 months (right).

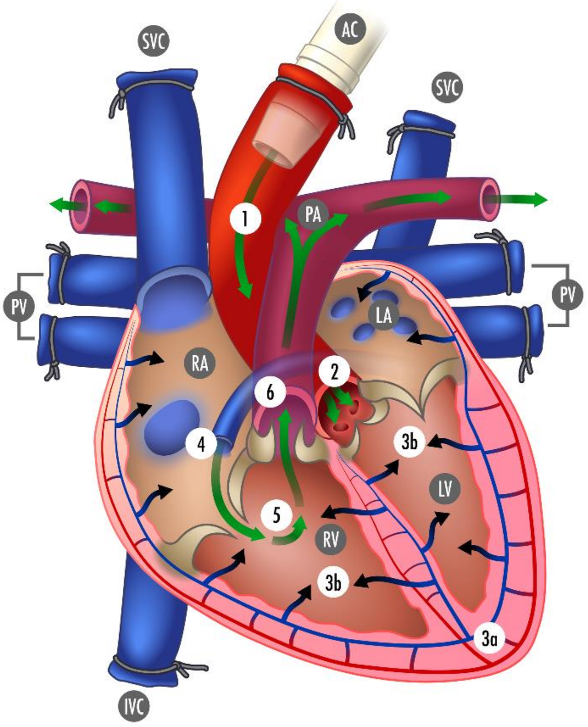

Figure S2. Perfusion flow pattern of the Langendorff method. The course and direction of perfusate flow through the heart takes the following pattern: 1. Perfusion starts from the aorta cannula in ascending aorta. 2. Coronary ostia to coronary arteries. 3a. Coronary arteries to myocardium, drainage to coronary veins (85-90% of flow), 3b. Direct coronary drainage into atria and ventricles via Thebesian vessels (10-15% of flow). 4. Venous drainage to right atrium and ventricle via coronary sinus 5. Right ventricle to pulmonary artery. 6. Effluent drains from right and left pulmonary arteries.

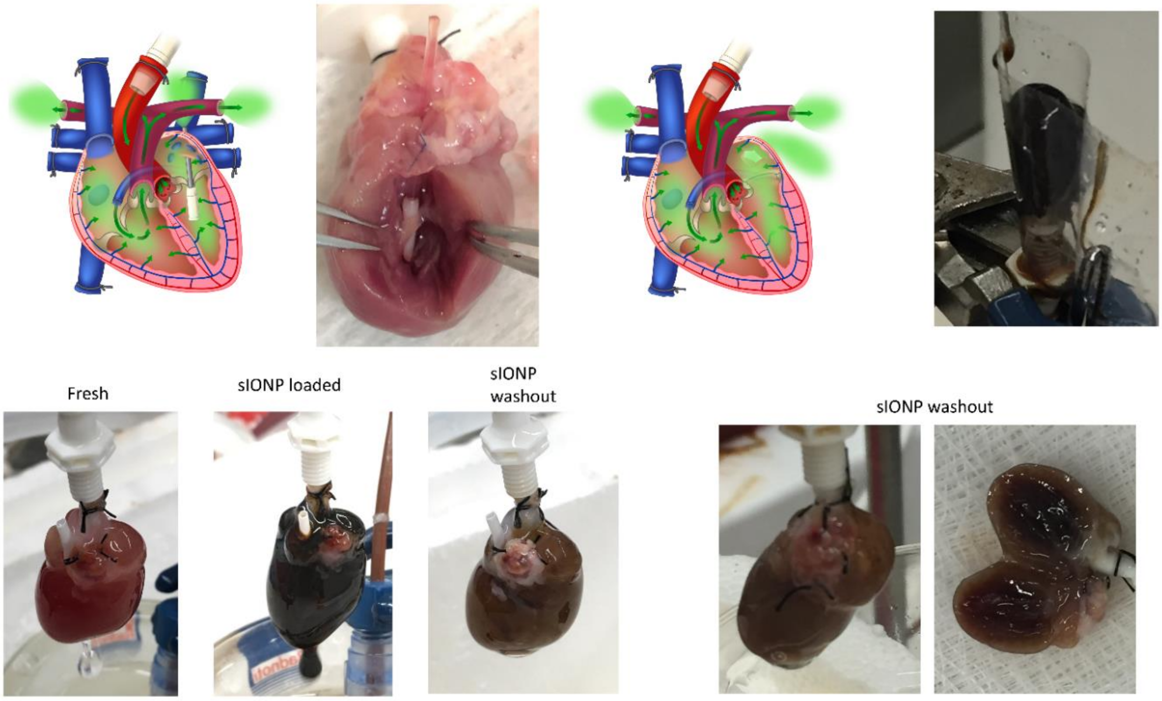

Figure S3. Abandoned cannulation methods investigated to relieve LV distension and sIONP washout. Alternative cannulation strategies which were tested and abandoned include the following: Insertion of a catheter through the mitral valve (left) and removal of the mitral valve and inversion of the heart during washout (right). Both methods resulted in significant sIONP residue in the sIONP washed-out hearts.

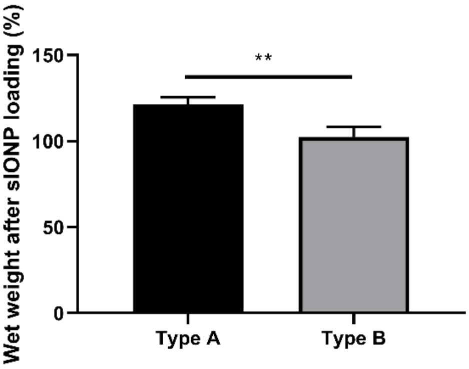

Figure S4. Wet weight of sIONP-loaded type A and type B hearts compared to the pre-loading wet weight (n=3). Type A perfusion resulted in a greater post-perfusion weight than type B perfusion which suggests accumulation of perfusate and sIONPs ($p < 0.01$).

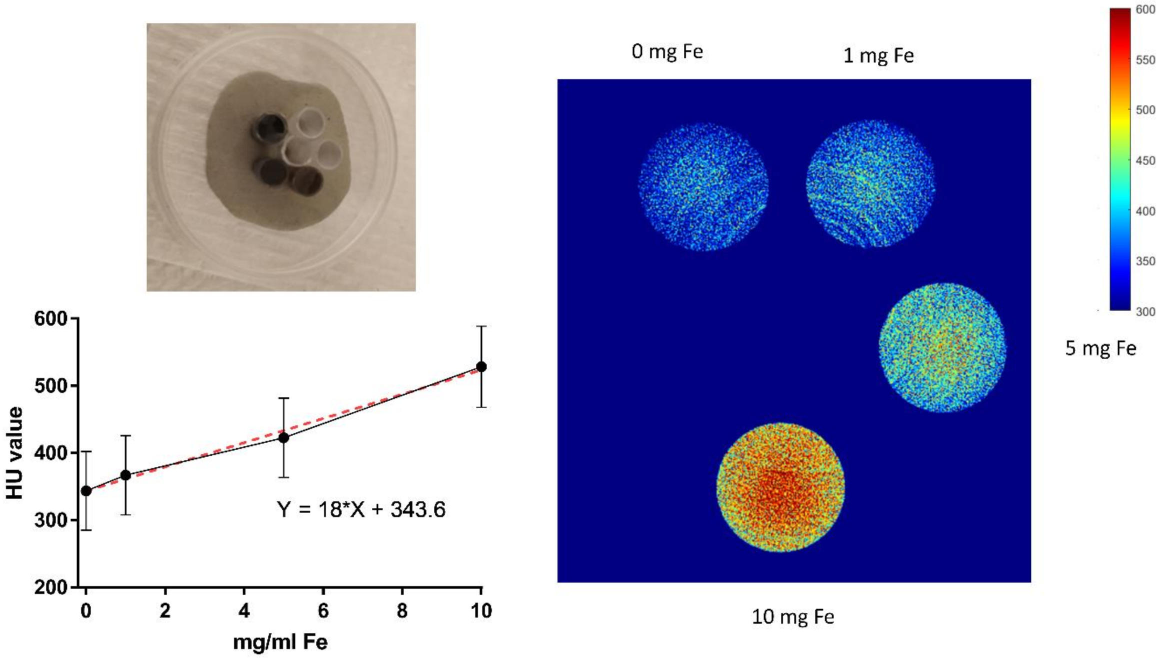

Figure S5. Micro CT calibration of sIONP concentration in VS55 at room temperature. The higher HU shows a linear relationship with higher sIONP concentration.

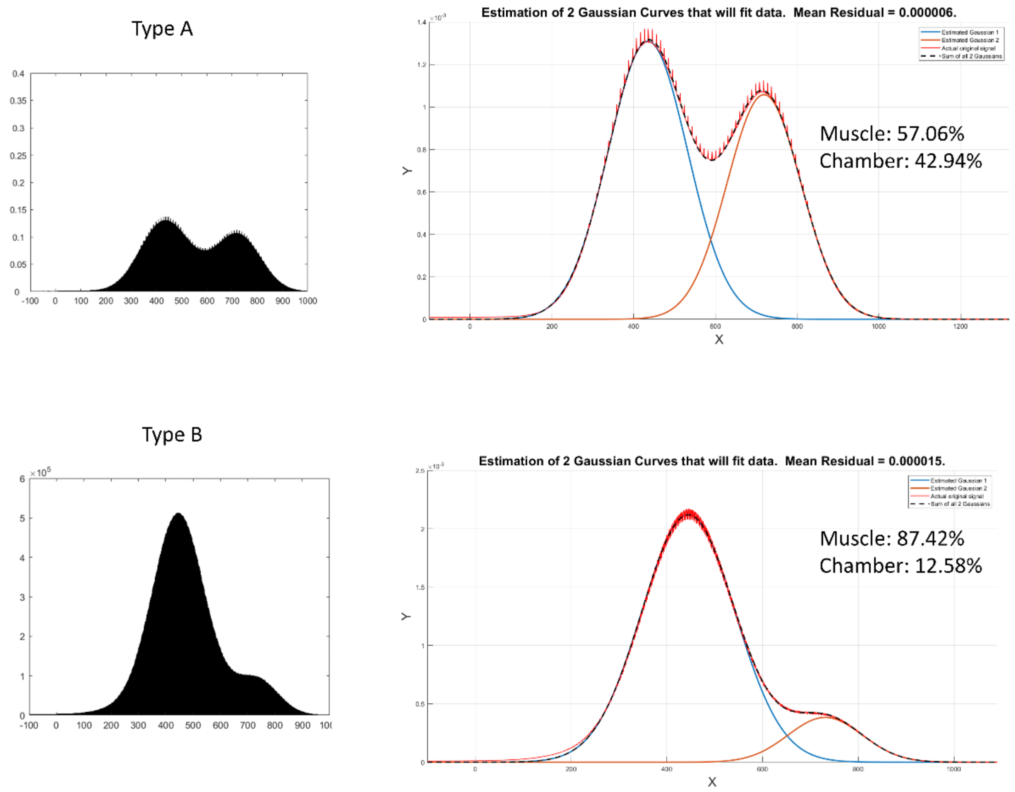

Figure S6. Analysis of sIONP-loaded type A and type B histograms by Gaussian curve fit. The overlapped histogram area was analyzed by Gaussian curve fit to obtain the muscle and chamber volume ratio by MATLAB.

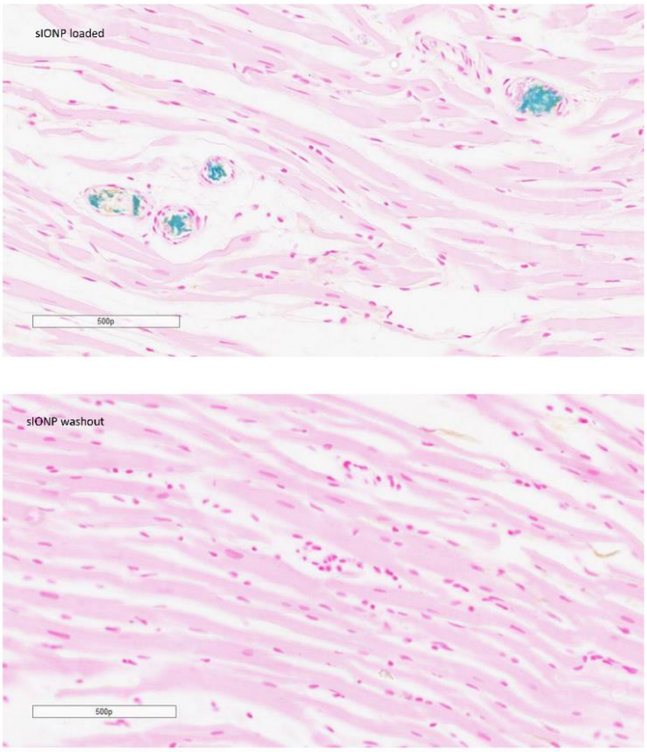

Figure S7. Prussian blue-stained sIONP-loaded (top) and washed-out type B hearts (bottom).

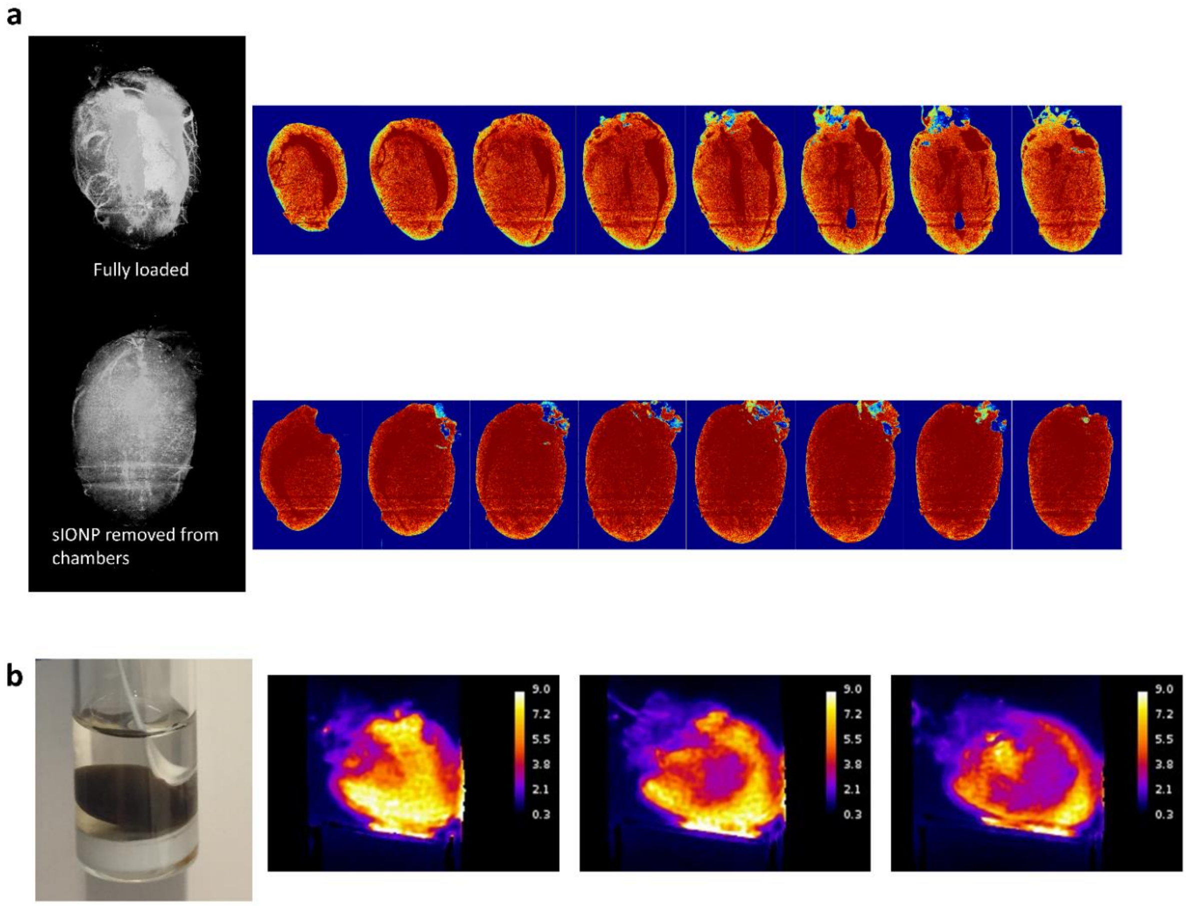

Figure S8. Imaging of sIONPs in fully loaded hearts and from hearts with sIONP removed from the chambers. a) µCT images of a heart before (top) and after sIONP removal from the chambers (bottom). b) SWIFT R1 map of a heart with sIONPs only in the myocardium. Scale bar is 1/s.

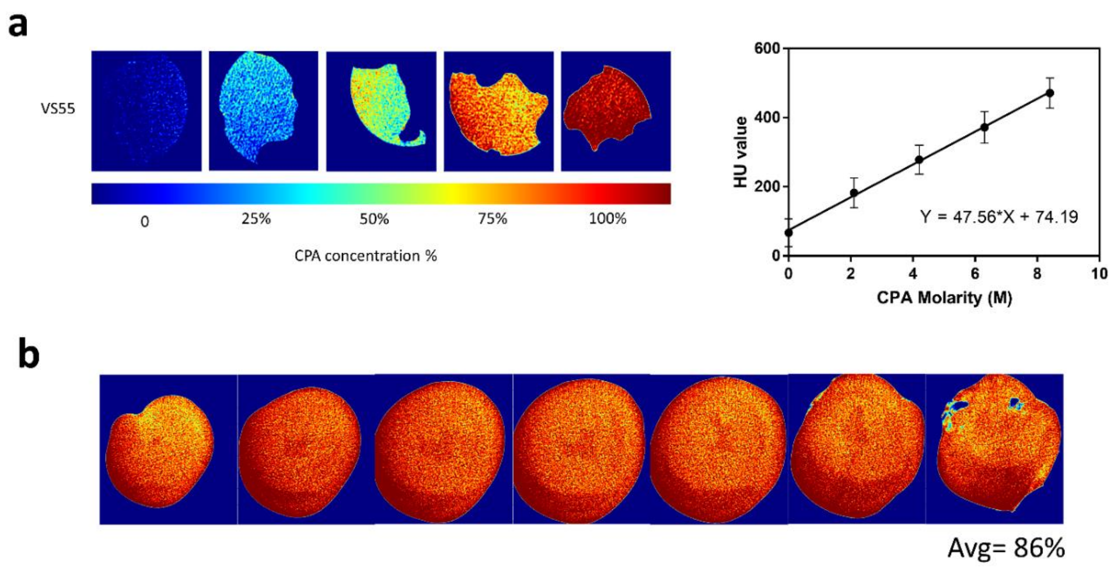

Figure S9. VS55 loading in type B perfused hearts. a) µCT calibration of VS55 concentration in heart tissue. The VS55 saturated heart tissues were prepared by placing in known VS55 concentration solutions for 2 weeks at 4°C. The incubation VS55 solutions were replaced twice during the incubation. b) µCT images of VS55 distribution in heart by the current perfusion method.

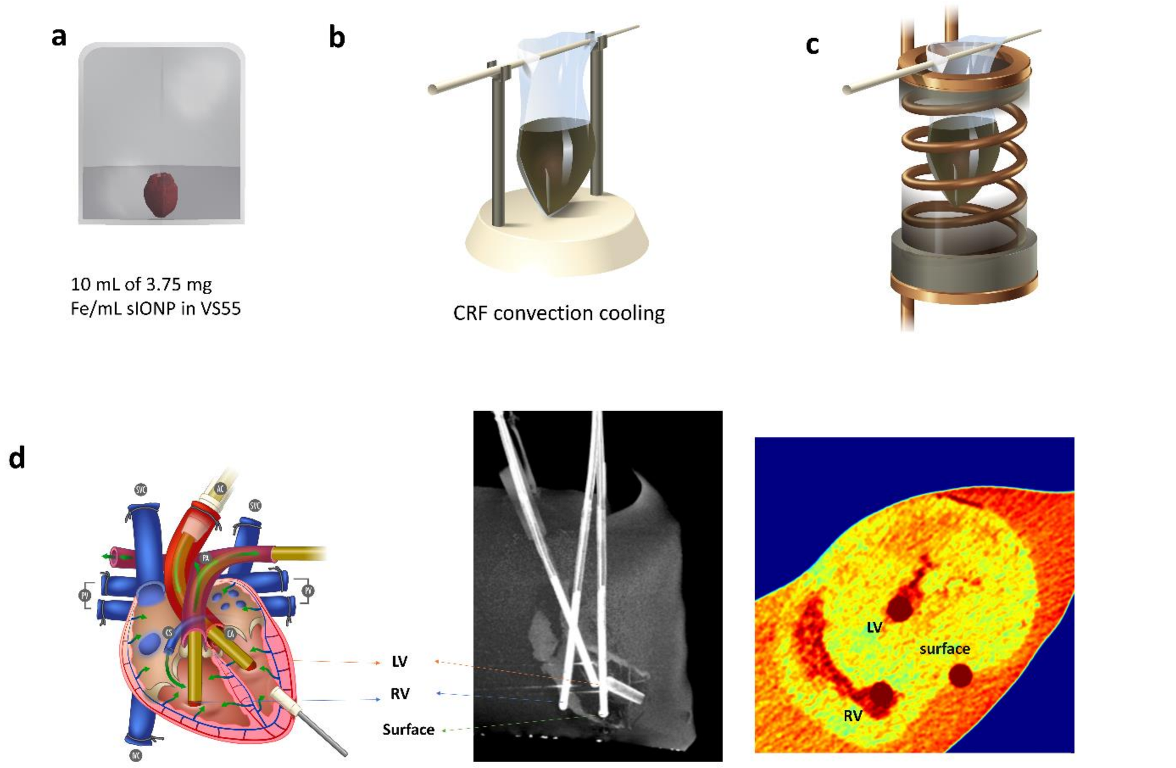

Figure S10. Cooling and nanowarming set up. a) Illustration of a rat heart in a 2 × 3 inch bag. b) Illustration of a sample bag cooled in a controlled rate freezer. c) Illustration of a sample rewarmed in a RF coil. d) Locations of the fiber optic probes in the heart.

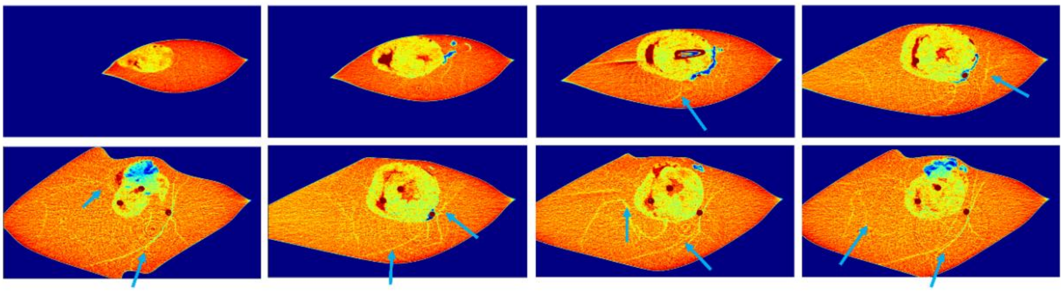

Figure S11. Micro CT images of a cracked heart in sIONP/VS55. Arrows indicate the cracks. This figure indicates the potential use of μCT for quality control in vitrified organs.

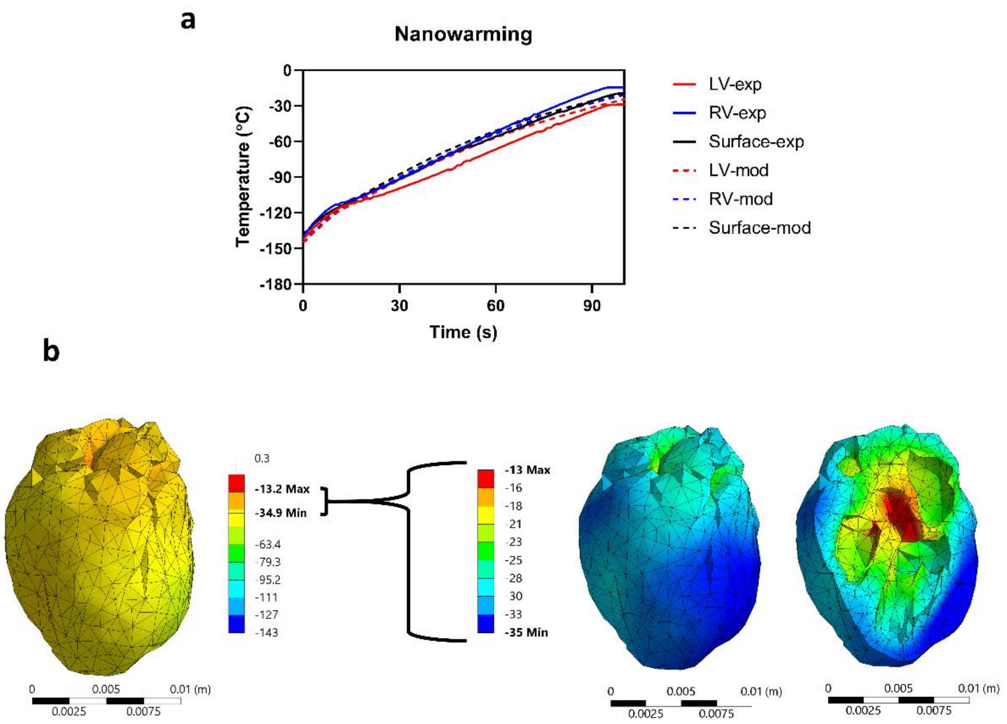

Figure S12. Modeling of nanowarming in the rat heart. a) Comparison of modeling and experimental data in a nanowarmed heart. b) Simulated cross-section of a nanowarmed heart. Legend: Con-LV (convectively cooled left ventricle), Con-RV (convectively cooled right ventricle), Con-surface (convectively cooled heart surface). NW-LV (nanowarmed left ventricle), NW-RV (nanowarmed right ventricle), and NW Surface (nanowarmed heart surface). Scale is shown in meters (m) below the organs in b.

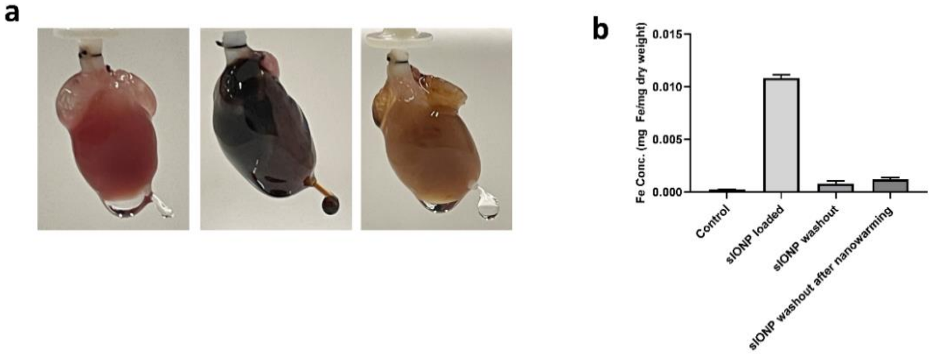

Figure S13. sIONP removal after sIONP/VS55 loading, vitrification and nanowarming. a) Photos of a heart pre-sIONP loading, post-sIONP loading, washout post vitrification and nanowarming. b) the Fe quantification of sIONP residue in the washed out heart post vitrification and nanowarming (control, n=7, sIONP loaded, n=5, sIONP washout, n=5, sIONP washout after nanowarming, n=3).

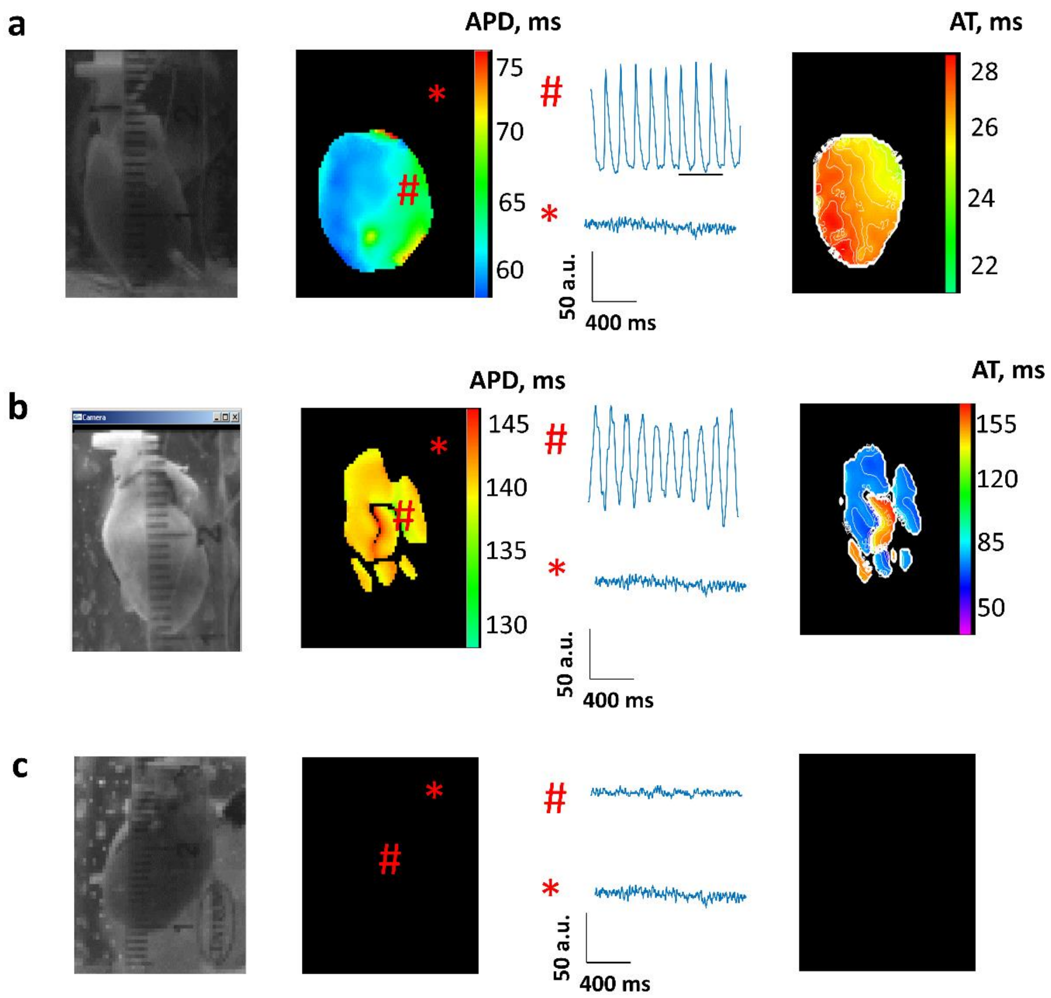

Figure S14. Optical mapping of fresh control (n=3) (a), nanowarmed (n=3) (b) and an electrically silent (c) hearts paced at BCL=120 ms. Left panels: raw images of the hearts with ruler. Middle panels: 2-dimentional (2D) action potential duration (APD) maps along with representative examples of traces taken from pixels of the heart (#) and outside of heart (*). Color bar indicates APD in ms. Mean APD for nanowarmed heart was significantly larger (139.05 ± 2.12 ms, $p<0.05$ by ANOVA) than for control heart (72.61 ±7.78 ms). Right panels: 2D isochronal maps of activation time (AT). Color bar indicates AT in ms.

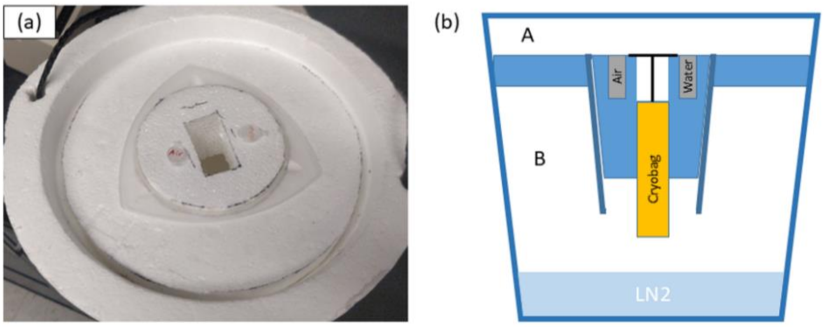

Figure S15. Micro CT setup for cryogenic samples. (a) Photo of μCT setup. (b) Schematic diagram, chamber A is at room temperature, chamber B is at LN2 vapor temperature (-150 °C). Note the presence of both air and water standards around the cryobag which help in the assignment of HU.

Table S1: Material properties used for computational analysis in the current study for sIONP/VS55 and Polyethylene as the cryobag material.

Material	Property	Value
VS55	Density, kg/m^3	$1078 - 0.43T$ [1]
	Specific heat, J/kg°C	Adapted from Etheridge et al.[2]
	Thermal conductivity, W/m°C	$(8.16 \times 10^{-4}) \times T + 4.40 \times 10^{-1} \quad -180 \le T \le -123$ [3]<br>$(4.59 \times 10^{-4}) \times T + 3.74 \times 10^{-1} \quad -96 \le T \le -77, -34 \le T \le 0$
Polyethylene	Density, kg/m^3	$931 - 0.49 \times T - 0.00091 \times T^2$ [4]
	Specific heat, J/kg°C	$1031 + 3.57 \times T - 0.014 \times T^2 - 6.1710 \times 10^{-5} \times T^3$ [5]
	Thermal conductivity, W/m°C	$0.39 + 5.2 \times 10^{-4} \times T$ [6]

[1] L. E. Ehrlich, G. M. Fahy, B. G. Wowk, J. A. Malen, Y. Rabin, *Journal of biomechanical engineering* **2018**, 140.

[2] M. L. Etheridge, Y. Xu, L. Rott, J. Choi, B. Glasmacher, J. C. Bischof, *Technology* **2014**, 2, 229.

[3] L. E. Ehrlich, Z. Gao, J. C. Bischof, Y. Rabin, *Plos one* **2020**, 15, e0238941.

[4] E. Hunter, W. G. Oakes, *Transactions of the Faraday Society* **1945**, 41, 49.

[5] U. Gaur, B. Wunderlich, *Journal of Physical and Chemical Reference Data* **1981**, 10, 119.

[6] R. Sheldon, S. K. Lane, *Polymer* **1965**, 6, 205.
